# Dynamics of Functional Traits and Molecular Regulation in the Vascular Cambium Across Different Ages of *Styphnolobium japonicum*

**DOI:** 10.3390/plants15152337

**Published:** 2026-07-29

**Authors:** Xuzhen Gao, Xingpeng He, Hao Wu, Shangjia Li, Shangyong Yin, Zhigang Xue, Yuan Liang, Huan Cao, Ran Wang, Bin Zhang, Jiawei Hao, Runmei Gao

**Affiliations:** 1 College of Forestry, Shanxi Agricultural University, Jinzhong 030801, China; gaoxuzhen56@163.com (X.G.); 18442834418@163.com (X.H.); 17696258091@163.com (H.W.); 18134910166@163.com (S.L.); 13734179449@163.com (S.Y.); x13383544750@163.com (Z.X.); 13990348608@163.com (Y.L.); ttw610610@163.com (H.C.); 18536494038@163.com (R.W.); 2 Taiyuan Institute of Cultural Heritage Conservation, Taiyuan 030025, China; 3 National Forestry and Grassland Administration’s Industrial Development Planning Institute, Beijing 100026, China; 13403365218@163.com (B.Z.); haojw93@163.com (J.H.)

**Keywords:** vascular cambium, *Styphnolobium japonicum*, old trees, longevity mechanism, transcriptome, metabolome

## Abstract

Understanding whether long-term vascular cambium vitality in ancient trees reflects progressive decline or adaptive reprogramming is central to grasping woody plant longevity. We performed an integrative analysis of functional traits, transcriptome profiles, and metabolomic landscapes of cambial zone enriched from 80-, 500-, and 1000-year-old *Styphnolobium japonicum* trees. With increasing tree age, the vascular cambium showed fewer cell layers, reduced thickness, and lower auxin, gibberellin, and IAA/ABA ratios, whereas bark thickness, malondialdehyde, abscisic acid, jasmonic acid, and salicylic acid contents increased. Transcriptomic and metabolomic analyses revealed that differentially expressed genes and metabolites were primarily enriched in the cell cycle, phytohormone signaling, and phenylpropanoid biosynthesis pathways. Specifically, genes associated with cell division were down-regulated in millennial trees, whereas phenolic acids, flavonoids, and lignin-related metabolites significantly accumulated. Piecewise structural equation modeling suggested associations among tree age, transcription factors, structural genes, metabolites, and cambial functional traits. These results indicate that cambial senescence is not a simple linear decay but a highly coordinated remodeling process, providing crucial evidence for delayed senescence in long-lived woody species.

## 1. Introduction

Understanding how vascular cambium activity is maintained across tree ages is central to explaining longevity in long-lived woody plants. As the lateral meristem that produces secondary xylem and phloem, the vascular cambium sustains radial growth, vascular renewal, and tissue repair throughout the life of a tree [1,2]. Increasing evidence suggests that exceptional longevity in trees is not explained simply by slow growth or low metabolism, but depends on the long-term stability of key meristematic tissues and their capacity to balance growth, maintenance, and environmental responses [3,4,5]. However, whether cambial vitality in aging trees over millennial timescales reflects progressive decline or adaptive remodelling remains unclear.

Age-related variation in cambial functional traits provides the most direct phenotypic evidence. Cambial activity and xylogenesis are age dependent, with older trees often showing shorter active periods, lower cell production, and altered anatomy [6]. In a 600-year-old *Ginkgo biloba*, for example, cambial cell layers decline with age, IAA decreases, and ABA increases, yet overall vitality is maintained [7]. Similar age-related contraction of cambial structure, together with persistence of anti-aging features, has also been reported in *Populus euphratica* [8]. More broadly, plant aging is commonly associated with reactive oxygen species accumulation, enhanced membrane lipid peroxidation, and reorganisation of antioxidant systems [9]. Hormonal regulation adds another layer of control: IAA, CK, and GA promote cambial division, expansion, and identity maintenance, whereas ABA, JA, and, in some contexts, SA are more closely linked to stress responses and aging regulation [10,11]. Together, these observations suggest that cambial aging in aging trees may involve a coordinated weakening of growth-related functions and reinforcement of defence- and maintenance-related functions, rather than simple linear decline.

Explaining such shifts requires integration of transcriptional regulation and metabolic remodelling. Cambial development is controlled by complex regulatory networks, and cell-type-specific transcriptomics in *Arabidopsis thaliana* have identified key modules centred on regulators such as *WOX4* and *KNAT1* [12]. In woody plants, direct transcriptomic analyses of cambium at different ages in *Cunninghamia lanceolata*, *Eucalyptus*, and *P. euphratica* have shown systematic reprogramming of genes related to cell division, cell wall remodelling, hormone pathways, and transcription factor networks [13,14]. Multi-omics work in old *Ginkgo* further revealed broad down-regulation of genes involved in cell division, expansion, and differentiation, together with sustained defence-related gene expression and protective secondary metabolism [7]. At the metabolic level, tree age can reshape phenylpropanoid metabolism and the accumulation of flavonoids, phenolic acids, and lignin-related compounds, suggesting reinforcement of structural protection and chemical defence in aging trees [15,16,17]. Yet most studies have examined transcriptomes or metabolomes separately, and an integrated causal framework linking tree age, regulatory genes, metabolite accumulation, and cambial functional traits is still lacking.

*S. japonicum* provides a suitable system to address these questions. It is a typical long-lived tree species in China with substantial ecological, cultural, and resource value, but existing work has focused mainly on germplasm resources, chloroplast genome variation, and secondary metabolite biosynthesis [18,19]. By contrast, little is known about how its vascular cambium maintains vitality over centennial to millennial timescales. The aging *S. japonicum* population in the Jinci region of Shanxi offers a rare natural system with a clear age gradient and relatively comparable environmental conditions.

Here, we used homologous vascular cambium samples from *S. japonicum* trees approximately 80, 500, and 1000 years old to quantify anatomical, stress-related physiological, and endogenous hormonal traits and integrated these data with transcriptomics, metabolomics, and piecewise structural equation modelling. We asked whether cambial aging in this species represents simple decline or a growth-to-defense/maintenance transition; whether this shift is associated with the contraction of cell-cycle and cell-expansion programmes, hormonal rebalancing, and enhanced phenylpropanoid/lignin metabolism; and whether core transcription factors mediate the direct and indirect effects of tree age on cambial functional traits. By addressing these questions, we aimed to establish an integrated framework linking tree age, transcription, metabolism, and functional traits and thereby provide new evidence for how cambial vitality is maintained in long-lived woody plants.

## 2. Results

### 2.1. Age-Related Shifts in Cambial Functional Traits

**Figure 1:**
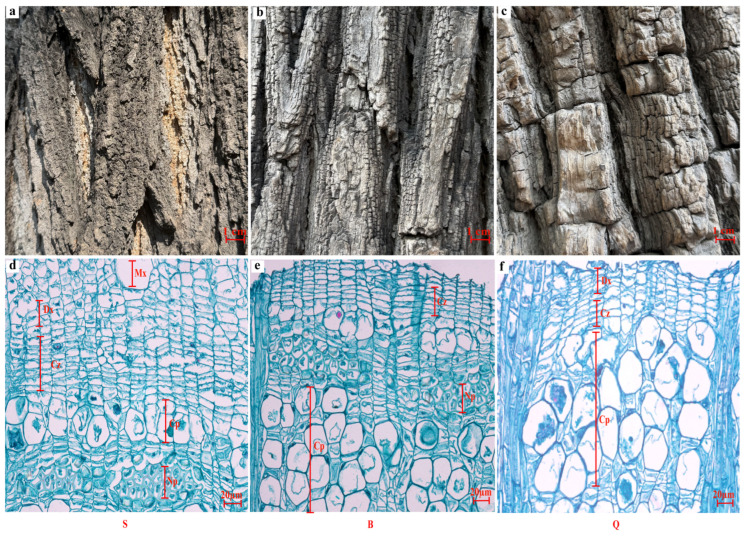
Morphological and anatomical features of *S. japonicum* trees of different ages: (**a**,**d**) 80-year-old trees (S group); (**b**,**e**) 500-year-old trees (B group); (**c**,**f**) 1000-year-old trees (Q group). The upper panels (**a**–**c**) show the external bark morphology of the respective age groups. The lower panels (**d**–**f**) are transverse sections of the stem tissues, illustrating the radial arrangement of nonconducting phloem (Np), conducting phloem (Cp), the cambial zone (Cz), differentiating xylem (Dx), and mature xylem (Mx). During sampling and tissue processing, mechanical separation along the cambial plane led to incomplete retention of xylem tissues in some sections, particularly in panels (**e**,**f**) from older trees. Therefore, these images are intended to illustrate the anatomical location of the cambial region and its adjacent tissues rather than to provide quantitative comparisons of tissue dimensions. Scale bars: (**a**–**c**) = 1 cm; (**d**–**f**) = 20 µm.

Anatomical observations revealed clear age-related changes in the cambial region of *S. japonicum* (Figure 1). Specifically, the 80-year-old trees had significantly more cambial cell layers (11.33 ± 1.53) than the 500-year-old (7.33 ± 1.15) and 1000-year-old (5.00 ± 1.00) trees, whereas no significant difference was detected between the latter two groups. Cambial thickness showed a similar pattern, declining from 118.98 ± 8.04 µm in the 80-year-old group to 79.58 ± 10.32 µm and 56.57 ± 9.23 µm in the 500- and 1000-year-old groups, respectively. Cambial thickness was significantly greater in the 80-year-old trees than in the two older groups, while the difference between the 500-and 1000-year-old trees was not significant.

**Figure 2:**
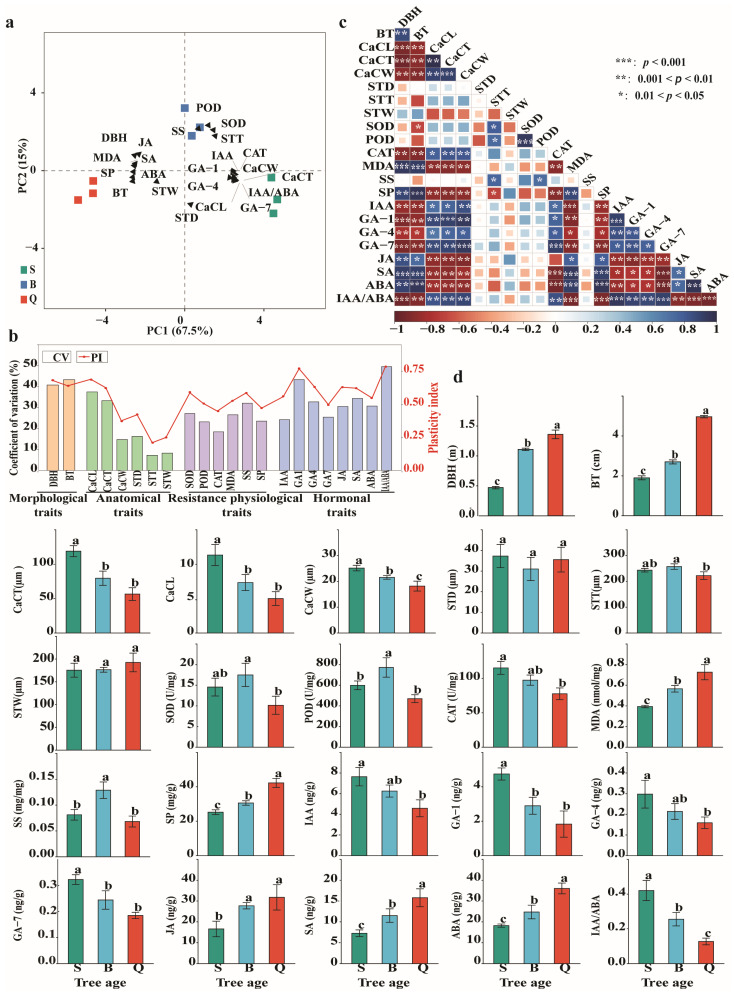
Analysis of vascular cambium functional traits in *S. japonicum* across different tree ages. (**a**) Principal Component Analysis (PCA) of functional traits. Black triangles (▲) indicate the loading vectors of key functional traits significantly contributing to PC1 and PC2. (**b**) Comparison of the PI and CV for the measured traits. (**c**) Correlation analysis among vascular cambium functional traits. (**d**) Developmental trends of vascular cambium functional traits in relation to tree age. Data shown were means ± standard deviations (n = 3). Different letters (a–c) above the bars indicate significant differences based on Tukey’s HSD test (*p* < 0.05).

A total of 82.5% of the variance was jointly explained by PC1 (67.5%) and PC2 (15.0%), which provided a distinct clustering of cambial traits across different tree ages (Figure 2a). Trait plasticity was substantial overall, with phenotypic PI values ranging from 0.2 to 0.8 and CVs from 7.5% to 49.5% (Figure 2b). Ten traits, including DBH, bark thickness, cambial cell layer number, cambial thickness, soluble sugars, GA-1, GA-4, JA, SA, and the IAA/ABA, exceeded the overall means for both PI and CV (PI_mean = 0.55; CV_mean = 28.39%), indicating high age sensitivity. Across all traits, CV was strongly positively associated with PI (CV = −11.05 + 71.90 PI, R^2^ = 0.92, *p* < 0.001).

Several traits increased significantly with age and were positively correlated with DBH, including bark thickness, MDA, soluble protein, ABA, and SA (Figure 2c,d). DBH increased from 47 cm in 80-year-old trees to 136 cm in 1000-year-old trees, bark thickness from 1.90 to 4.97 cm, and MDA from 0.39 to 0.73 nmol/mg. An inverse relationship with DBH was observed for cambial thickness, cell layer number, cell width, CAT, and hormones (IAA, GAs, and the IAA/ABA). Cambial thickness decreased from 118.98 to 56.57 µm, cambial cell layers from approximately 11.33 to approximately 5, and the IAA/ABA from 0.42 to 0.13.

### 2.2. Age-Related Transcriptomic Reprogramming

#### 2.2.1. RNA-Seq Overview

RNA-seq of vascular cambium from 80-year-old (S), 500-year-old (B), and 1000-year-old (Q) trees generated 102.74 Gb of clean data, with more than 6.44 Gb per sample. GC content ranged from 44.45% to 45.04%, Q30 values exceeded 96.8%, and mapping rates were higher than 95% in all libraries, indicating high-quality transcriptomic data.

#### 2.2.2. Functional Enrichment of DEGs

**Figure 3:**
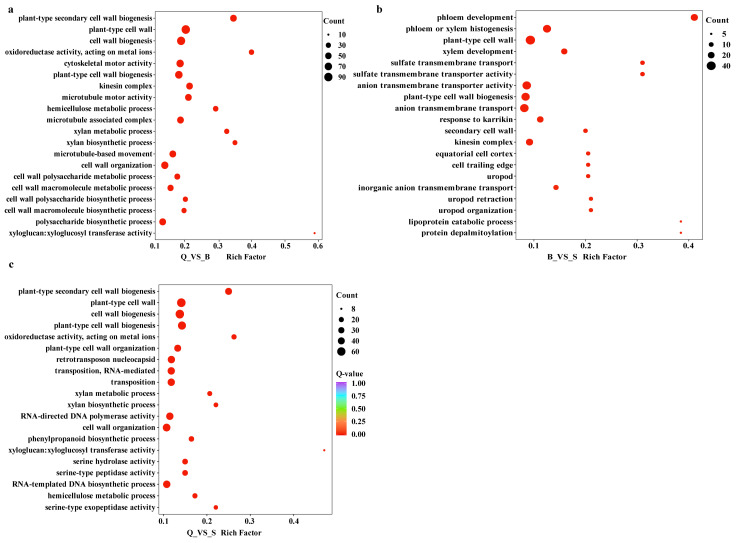
GO enrichment analysis of differentially expressed genes (DEGs) across three pairwise comparison groups. The analysis compares three growth stages: (**a**) Q_vs_B, (**b**) B_vs_S, and (**c**) Q_vs_S.

Transcriptomic analysis identified a total of 9079 DEGs associated with increasing tree age, with down-regulated genes consistently outnumbering up-regulated ones across all comparison groups (Table S1). GO enrichment analysis showed that DEGs were mainly associated with cell wall organisation and biosynthesis, vascular differentiation, and secondary metabolism. In B_vs_S, DEGs were enriched primarily in cell wall organization and biogenesis, secondary metabolism, and vascular tissue differentiation. In Q_vs_B, enriched terms included cell wall biosynthesis, secondary cell wall formation, hemicellulose and xylan metabolism, phenylpropanoid metabolism, and lignin biosynthesis. Q_vs_S showed the broadest divergence, with enrichment in cell wall formation, vascular development, and secondary metabolism (Figure 3).

KEGG analysis assigned 1221 DEGs to 202 pathways, mainly related to structural formation, secondary metabolism, and signalling. The most significantly enriched pathway was Motor proteins (102 genes, 8.35%), followed by Plant hormone signal transduction (80 genes, 6.55%), Plant-pathogen interaction (55 genes, 4.50%), and Amino sugar and nucleotide sugar metabolism. B_vs_S was enriched mainly in sugar metabolism, hormone signalling, and phenylpropanoid metabolism, whereas Q_vs_B showed the strongest enrichment overall, particularly for cell wall polysaccharide metabolism, secondary metabolism, and signalling. Q_vs_S additionally involved a broader set of secondary metabolic and signalling pathways.

### 2.3. Age-Related Metabolomic Reprogramming

#### 2.3.1. Identification and Analysis of DAMs

**Figure 4:**
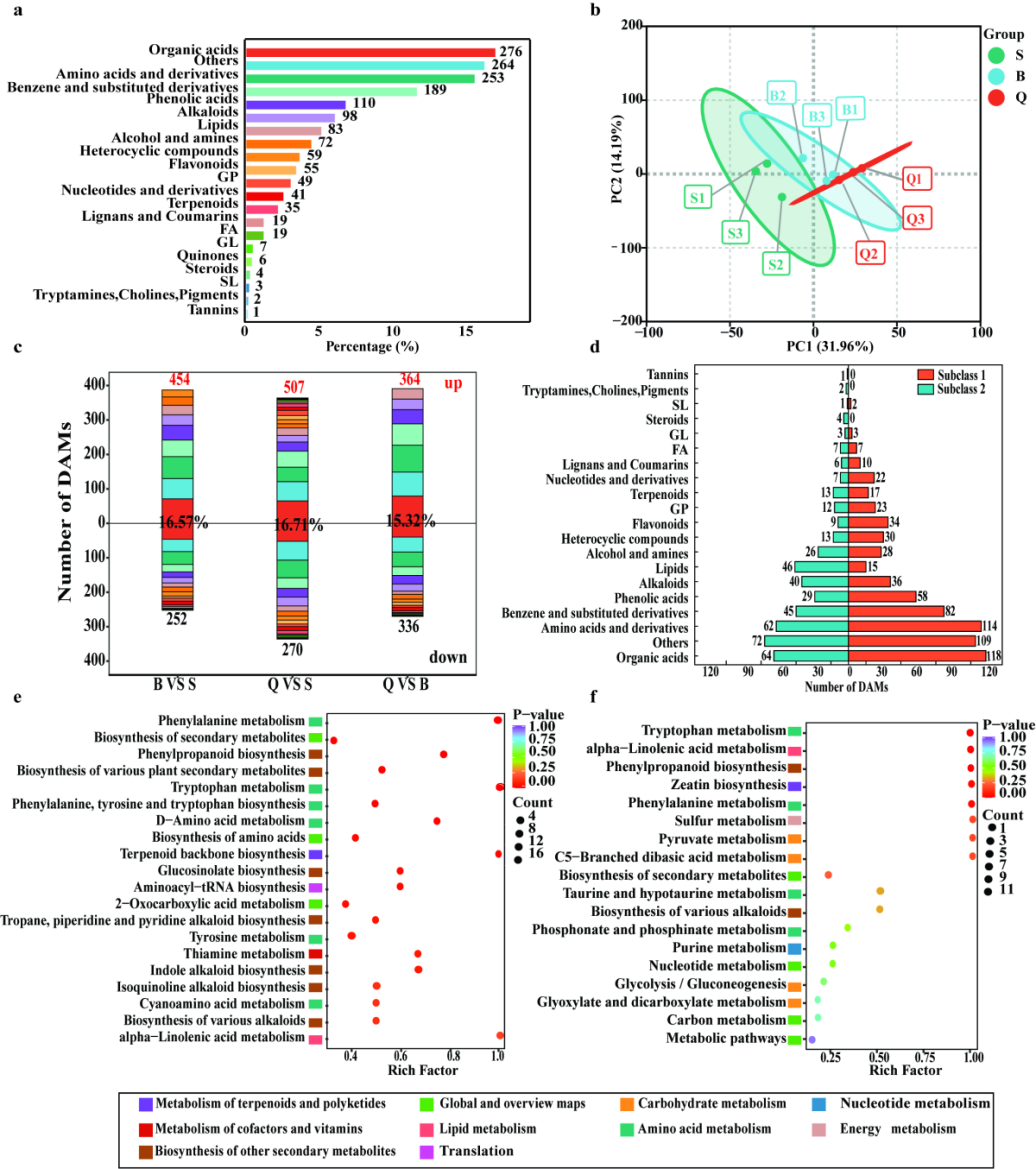
Dynamic metabolome analysis of *S. japonicum* vascular cambium across different tree ages. (**a**) Classification of the 1645 detected metabolites. (**b**) PCA plot based on all detected metabolites. (**c**) Total number of differentially abundant metabolites (DAMs) across different comparison groups; color coding follows the classifications in panel (**a**). (**d**) All DAM relative abundances categorized by chemical type within Subclass 1 and Subclass 2. (**e**,**f**) KEGG enrichment analysis of DAMs in Subclass 1 (**e**) and Subclass 2 (**f**). Colored squares indicate the superior classification category for each metabolic pathway.

A total of 1645 metabolites were identified and categorized into 21 primary classes in the metabolome analysis of nine samples (Figure 4a). PCA revealed an age-associated distribution pattern in the metabolomic profiles, with PC1 and PC2 explaining 31.96% and 14.19% of the total variance, respectively. Although the 500-year-old and 1000-year-old samples were not completely resolved in the unsupervised PCA plot, the 500-year-old samples were broadly positioned between the 80-year-old and 1000-year-old groups, suggesting an intermediate metabolic profile (Figure 4b). ANOSIM further confirmed significant differences in overall metabolomic composition among the three age groups (R = 0.6214, *p* = 0.018). In addition, OPLS-DA provided complementary visualization of the metabolomic discrimination among age groups (Figure S1).

A total of 1170 DAMs were identified across pairwise comparisons. Organic acids were the most abundant DAM class in all three comparisons (117–119 metabolites; 15.32–16.71%; Figure 4c). In B_vs_S and Q_vs_S, most metabolite classes were dominated by increases, whereas in Q_vs_B, up-and down-accumulated metabolites were more balanced. The major shifts involved organic acids, amino acids and derivatives, benzenes and substituted derivatives, phenolic acids, and subsets of lipids and secondary metabolites. Specifically, several flavonoid- and terpenoid-related metabolites, including ethyl salicylate, 5-methyl-7-methoxyisoflavone, icaritin, phenol glucuronide, and quercetin 3-O-(6″-acetyl-glucoside), accumulated strongly in the 1000-year-old cambium.

#### 2.3.2. Dynamic Metabolomic Analysis Across Ages

K-means clustering partitioned all DAMs into two subclasses: Subclass 1 (708 DAMs), which increased with age, and Subclass 2 (462 DAMs), which decreased with age. Organic acids were dominant in both subclasses, whereas lipids were more enriched in Subclass 2 and flavonoids in Subclass 1 (Figure 4d).

Pathway enrichment showed that Subclass 1 was dominated by secondary metabolism and amino acid metabolism, with significant enrichment of Phenylalanine metabolism, Phenylpropanoid biosynthesis, and Tryptophan metabolism (*p* < 0.05; Figure 4e). Subclass 2 was significantly enriched in Tryptophan metabolism, alpha-Linolenic acid metabolism, Phenylpropanoid biosynthesis, Phenylalanine metabolism, and Zeatin biosynthesis (Figure 4f). Differential abundance score analysis further showed that most secondary metabolic pathways increased with age, especially phenylpropanoid biosynthesis, phenylalanine metabolism, and biosynthesis of plant secondary metabolites (Figure S2). Overall, age-related metabolic remodelling was concentrated in phenylpropanoid and hormone biosynthetic pathways.

### 2.4. Integrated Multi-Omics Analysis

Integrated transcriptomic and metabolomic analyses highlighted cell wall construction and associated metabolic pathways as major axes of age-related cambial reprogramming. GO and KEGG analyses of the transcriptome showed pronounced divergence in genes related to cell wall formation and associated metabolism among age groups. Joint enrichment of DEGs and DAMs identified recurrent pathways, including Phenylpropanoid biosynthesis, Phenylalanine, tyrosine, and tryptophan biosynthesis, alpha-Linolenic acid metabolism, Phenylalanine metabolism, and Zeatin biosynthesis (Figure S3). We therefore focused on cell-cycle regulation, cell division/differentiation, phenylpropanoid-lignin metabolism, and plant hormone pathways.

#### 2.4.1. Cell Division and Expansion Programmes Decline with Age

**Figure 5:**
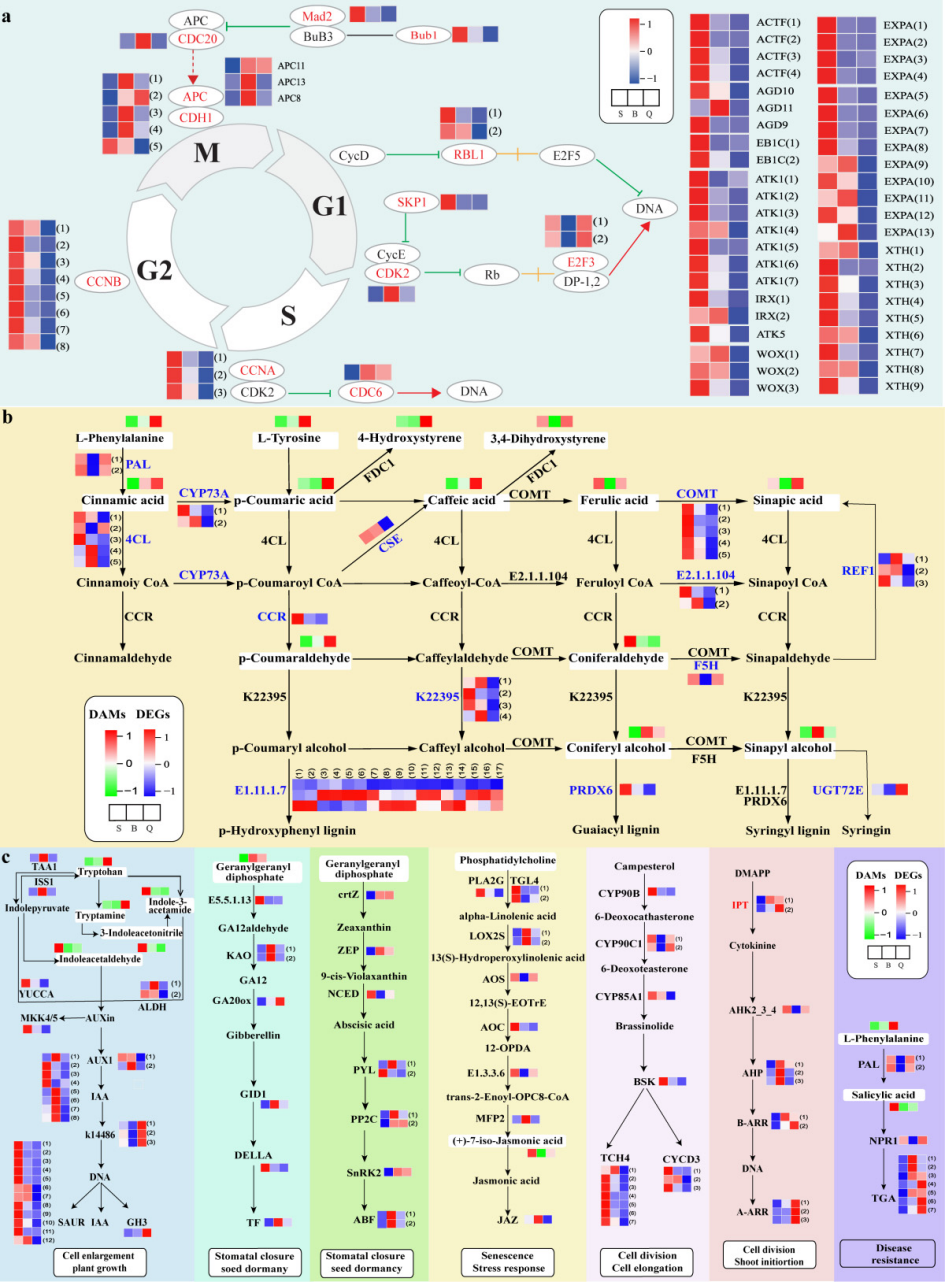
Expression profiles of key biological pathways in the vascular cambium of *S. japonicum* across different tree ages. (**a**) Expression patterns of genes involved in cell division and differentiation. Detailed expression values and fold changes are provided in Supplementary Table S2. (**b**,**c**) Integrated analysis of DEGs and DAMs within (**b**) the phenylpropanoid biosynthesis pathway and (**c**) various hormone pathways. In all heatmaps, the color gradient from blue to red represents transcript or abundance levels ranging from low to high. Gene symbols are indicated in blue font; see Tables S3–S5 for detailed gene information.

Most core cell-cycle regulators showed strong age-dependent expression patterns (Figure 5a). Positive regulators of cell division, including *CycA*, *CycB*, and *Bub1*, were generally down-regulated with age. Some genes, including *Cdc*, *CDK*, *APC13*, and *APC8D*, were more highly expressed in the 500-year-old samples, whereas *APC11* and *CDC6* remained elevated from 500 to 1000 years. Additional genes associated with cell division and expansion, including *WOX*, *actin*, *AGD*, *EB1C*, *ATK*, *expansins*, *cellulose synthase A catalytic subunits*, and *xyloglucan endotransglycosylases*, were expressed at lower levels in the 1000-year-old cambium than in the 80-year-old cambium. Overall, genes involved in cell-cycle regulation, cell division, and cell expansion showed a clear downward trend with age.

**Figure 6:**
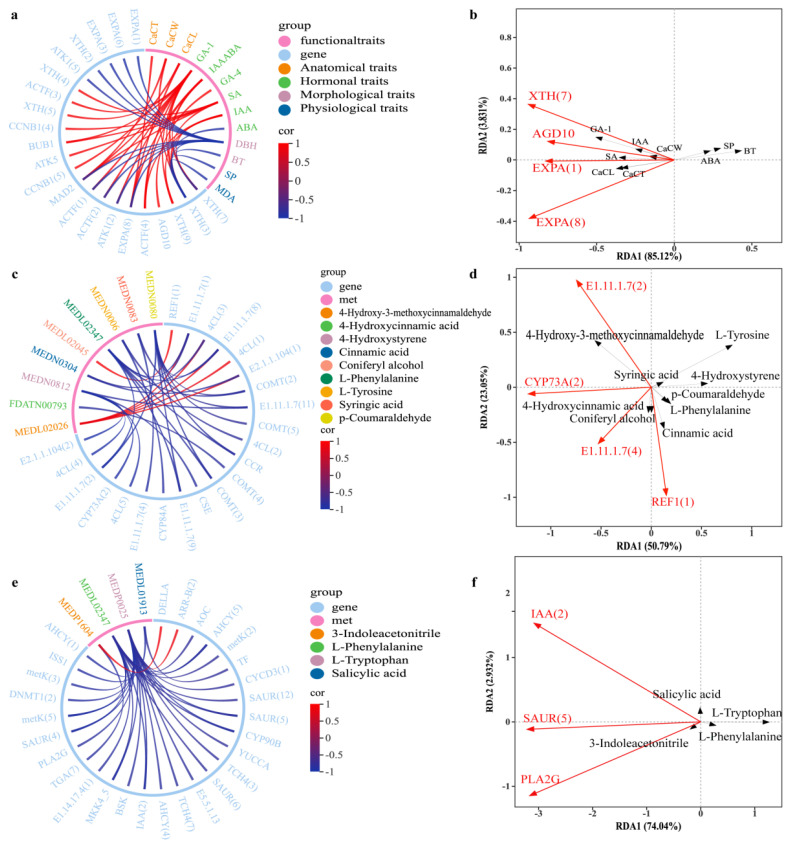
Correlation and RDA of molecular and functional components in *S. japonicum*. (**a**,**c**,**e**) Correlation analysis and (**b**,**d**,**f**) RDA of DEGs, DAMs, and functional traits across three biological contexts: (**a**,**b**) cell division and differentiation pathways versus functional traits; (**c**,**d**) general phenylpropanoid and lignin biosynthesis pathways; and (**e**,**f**) hormone pathways.

Correlation analysis between cell division/differentiation-related DEGs and cambial traits identified 31 significant positive and 16 significant negative associations (|r| > 0.80, *p* < 0.05), involving 13 traits and 22 DEGs (Figure 6a). Similarly, RDA identified *EXPA(1)*, *EXPA(8)*, *XTH(7)*, and *AGD10* as key genes associated with age-related changes in cambial cell division, expansion, and differentiation (Figure 6b).

#### 2.4.2. Phenylpropanoid and Lignin Metabolism Are Reinforced with Age

The phenylpropanoid biosynthetic pathway contained 14 DAMs (Figure 5b). Apart from phenylalanine and tyrosine, 12 of these metabolites (85.7%) were phenolic acids. Several metabolites, including 3-p-coumaroylquinic acid, 4-hydroxycinnamic acid, caffeic acid, 4-hydroxystyrene, coniferyl alcohol, sinapyl alcohol, and p-coumaraldehyde, increased significantly with age. In the same pathway, 58 DEGs encoding 13 enzymes were identified, including E1.11.1.7, *4CL*, *COMT*, K22395, *REF1*, *CYP73A*, E2.1.1.104, *PAL*, *CCR*, *CSE*, *CYP84A*, *PRDX6*, and *UGT72E*. Notably, despite marked accumulation of phenylpropanoid- and lignin-related metabolites in the 1000-year-old trees, several key enzyme genes, including E1.11.1.7, *4CL*, *COMT*, K22395, *CYP73A*, E2.1.1.104, *CCR*, *CSE*, and *PRDX6*, were significantly down-regulated. Numerous genes were differentially expressed in this pathway (Figure 6c), and subsequent correlation analysis identified 28 pairs of significant negative correlations and six pairs of significant positive correlations, involving nine DAMs and 22 DEGs. In addition, E1.11.1.7(2), E1.11.1.7(4), *CYP73A(2)*, and *REF1(1)* as key genes associated with age-related shifts in phenylpropanoid and lignin metabolism (Figure 6d).

#### 2.4.3. Hormone Pathways Are Extensively Reprogrammed Across Tree Ages

Hormone biosynthesis metabolic pathways also showed strong age-related reprogramming (Figure 5c). In the IAA pathway, the 80-year-olds were characterised by higher levels of IAM- and IAN-branch metabolites and higher expression of *YUCCA*, *MKK4/5*, and *AUX1*, whereas the 1000-year-olds showed stronger changes in the tryptamine-dependent route. In the cytokinin pathway, *IPT*, *AHP*, and *B-ARR* were more highly expressed in the 500- and 1000-year-old, and *A-ARR* was further up-regulated in the 1000-year-old. GA- and BR-related pathways were also age dependent: BR-related genes such as *CYP* and *TCH4* were higher in the 80-year-old, GA-related genes such as *KAO* and *GID1* were higher in the 500-year-old, whereas both pathways were broadly down-regulated in the 1000-year-old. By contrast, the ABA pathway showed greater precursor accumulation and higher expression of regulators such as *SnRK2* in the 500- and 1000-year-old trees. SA-and JA-related pathways also differed among age groups: relevant metabolites accumulated more strongly in the 80-year-old, whereas regulators such as *TGA* were more active in the 500 and 1000-year-old.

The results showed that four DAMs were significantly associated with 20 DEGs (|r| > 0.80 and *p* < 0.01), with two pairs showing significant positive correlations and 23 pairs showing significant negative correlations (Figure 6e). Similarly, RDA indicated that *PLA2G*, *SAUR*(5), and *IAA*(2) as key genes associated with age-related changes in hormone metabolism (Figure 6f).

### 2.5. Core Transcription Factors Associated with Tree Age

**Figure 7:**
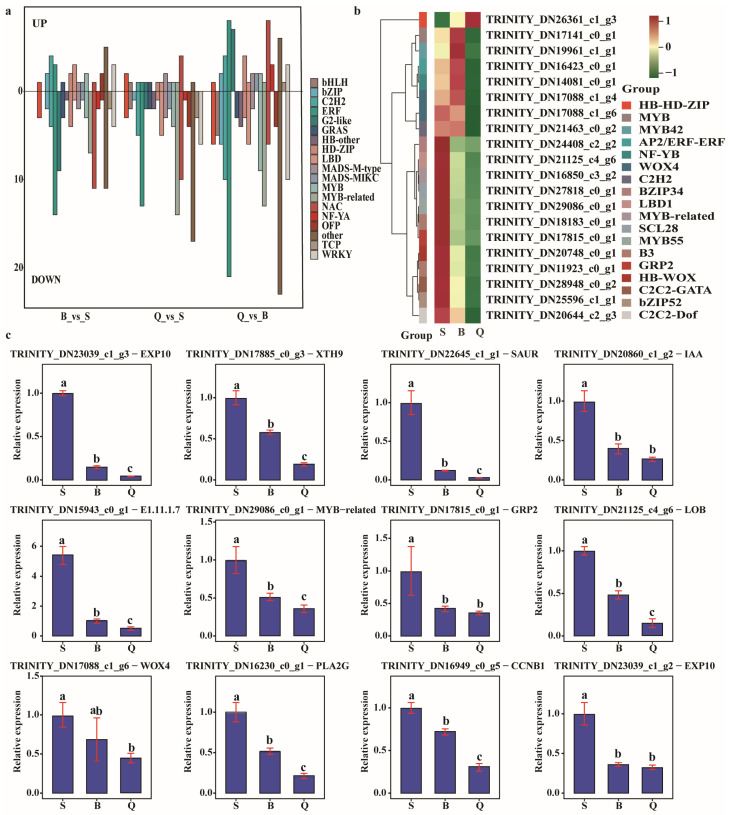
Identification and validation of key TFs in *S.japonicum*. (**a**) Distribution of upregulated and downregulated TFs categorized by family across different comparison groups. (**b**) Heatmap illustrating the expression profiles of the top 20 differentially expressed TFs. (**c**) RT—qPCR validation of 12 selected DEGs from the transcriptome data. The corresponding RNA-seq expression values for these genes have been provided in Supplementary Table S6. Different lowercase letters (a, b, c) above the bars indicate significant differences between groups (one-way ANOVA with Tukey’s HSD post-hoc test, *p* < 0.05).

Among 1619 detected transcription factors (TF), 284 were differentially expressed and belonged to 43 TF families. The most abundant families were ERF (14.8%), MYB-related (8.1%), and NAC (8.1%), all of which are closely linked to hormone signaling, stress responses, vascular development, and lignin/cellulose biosynthesis. Down-regulated differentially expressed transcription factors (DETFs) accounted for the majority of DETFs in each comparison and reached 84.03% in Q_vs_S (Figure 7a).

Hub TFs were identified based on correlation analysis of 124 DEGs and DETFs from age-related metabolic pathways (|r| > 0.80, FDR < 0.05). The top 20 candidate TFs were retained. Many showed strong age dependence (Figure 7b). Among these TFs, *SjLBD1*, *SjMYB55*, *SjSCL28*, *SjbZIP52*, *SjB3*, *SjGRP2* and *SjbZIP34* declined progressively with age. RT-qPCR validation of 12 randomly selected DEGs matched the RNA-seq trends, confirming the reliability of the transcriptomic data (Figure 7c).

### 2.6. Responses of Functional Traits, Metabolites and Gene Expression to Tree Age

**Figure 8:**
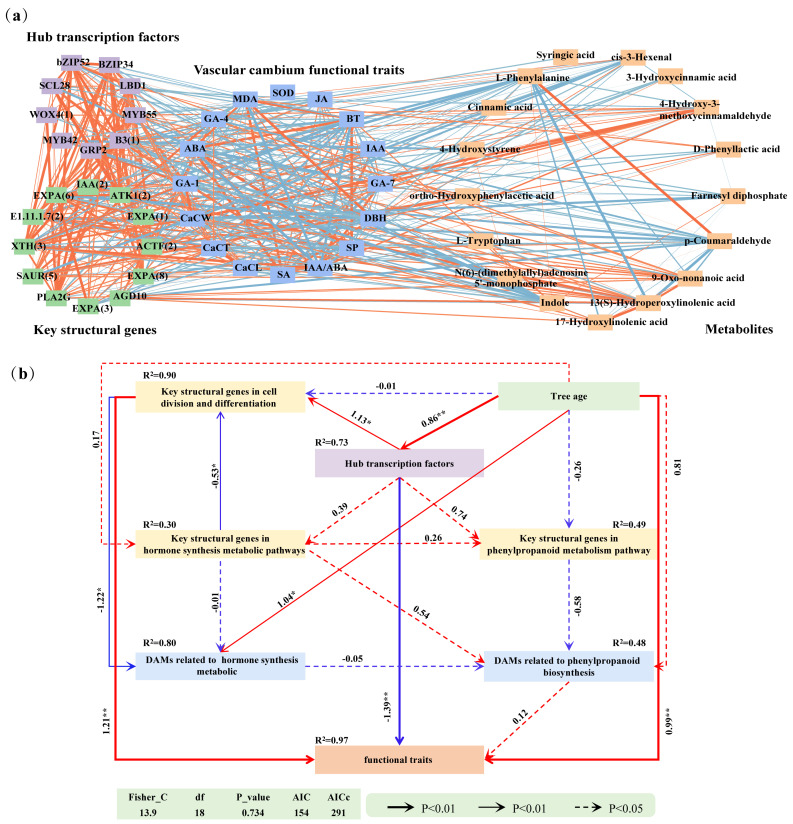
Responses of functional traits, metabolites, and genes to tree age of *S. japonicum*. (**a**) Pearson correlation network. Orange represents positive correlation; blue represents negative correlation. The line thickness is proportional to the size of the correlation coefficient. (**b**) The segmented structural equation model revealed the causal relationships between tree age factors, core transcription factors, key structural genes, metabolites, and functional traits. The red arrow represents the positive path, and the blue arrow represents the negative path. Asterisks indicate significance levels of the path coefficients: * *p* < 0.05, ** *p* < 0.01.

Strong correlations were among hub TFs, cell division/differentiation, key structural genes and metabolites involved in hormone metabolism and phenylpropanoid metabolism pathways, and cambial functional traits a correlation network (|r| > 0.80, *p* < 0.01) (Figure 8a). For example, four hub TFs were significantly correlated with indole, three with p-coumaraldehyde, and two with L-phenylalanine. Nine hub TFs were significantly correlated with *XTH(3)*, and five with *SAUR*(5) and MDA.

Ultimately, piecewise structural equation modelling further resolved the causal relationships among tree age, hub TFs, cell division/differentiation, key genes, and metabolites involved in hormone metabolism and phenylpropanoid metabolism pathways, along with cambial functional traits (Figure 8b). Tree age had significant direct effects on hormone-related metabolites and functional traits and indirect effects mediated through hub TFs and downstream genes related to cell division and differentiation. Hub TFs also exerted direct effects on cambial traits. In addition, key genes involved in cell division and differentiation showed negative feedback relationships with DAMs in hormone pathways and directly influenced cambial functional traits.

## 3. Discussion

### 3.1. Cambial Aging Reflects Coordinated Remodelling

Rather than following a simple trajectory of progressive deterioration, cambial aging was associated with coordinated changes in anatomical, hormonal, and metabolic traits. We found that cambial cell layer number and cambial thickness declined with age. The 80-year-old group had significantly cambial thickness than both older groups, whereas no significant difference was observed between the 500- and 1000-year-old groups. Older *S. japonicum* trees also showed thicker bark, higher ABA, JA, and SA contents, and alteration in cambial physiological status. Similar patterns have been described in *Ginkgo* and *P. euphratica*, where older cambium showed contraction of growth-related structures but retention of vitality and anti-aging molecular features [7,8]. This is consistent with broader plant aging theory, which documents longevity not as escape from aging, but as prolonged maintenance of essential meristematic functions under changing physiological constraints [20,21,22]. Therefore, the nature of cambial aging in aging trees may involve a gradual reallocation of functional priorities rather than terminal systemic collapse.

The intermediate position of the 500-year-old trees across functional traits, transcriptional profiles, and metabolite patterns suggested that cambial aging proceeds as a gradual remodelling process rather than an abrupt shift, in which high-growth programmes progressively attenuate, while stress and secondary metabolism associated signatures become more prominent. Such a transitional configuration fits the idea that long-lived trees may maintain persistence through regulated resource reallocation rather than simple growth cessation or catastrophic decline. It also aligns with wider ecological observations that trajectories of tree growth and aging are rarely linear and are shaped by resource allocation, modular persistence, and long-term environmental buffering [23,24].

### 3.2. Contraction of Growth Programs and Hormonal Rebalancing Underlie Cambial Aging

At the molecular level, cambial aging was associated with reduced expression of genes involved in cell division and differentiation. Down-regulation of *CycA*, *CycB*, *Bub1*, *XTH*, and cellulose synthesis-related genes closely matched the decline in cambial cell layer number, thickness, and cell width. These patterns are consistent with attenuation of the regulatory machinery required to sustain proliferation and expansion. Comparable age-related reductions in xylogenesis and cambial output have been reported in *Ginkgo*, *the Tibetan Plateau*, and *Cryptomeria japonica* [7,25,26]. Nevertheless, the retention of some regulatory activity in intermediate-age trees suggested that proliferative capacity was selectively retained at a lower level, which may be sufficient to preserve cambial continuity over long timescales.

This attenuation of growth-associated programmes was accompanied by pronounced hormonal rebalancing. Declining IAA and GA, increasing ABA, and a sharp reduction in the IAA/ABA ratio indicated that the regulatory environment of the cambium shifts away from promoting division, expansion, and differentiation toward a more growth-constrained and stress-responsive physiological state. This is strongly supported by current models of cambial regulation, in which auxin, CK, GA, and BR sustain cambial activity, whereas ABA integrates seasonal signalling and stress-related restraint [27,28,29]. Recent functional studies in *Poplar* further demonstrated that ABA was directly connected to auxin-mediated cambial activity through the *AREB4/AREB13-VCM1/VCM2-PIN5b* module, while JA and CK were coupled through *PagJAZ5*, and *WOX4*-dependent cambial activity was constrained by ubiquitin-mediated control and epigenetic regulation [30,31,32,33]. GA-dependent cambium re-establishment after girdling provided additional evidence that hormonal signalling continued to shape secondary vascular development in a highly context-dependent manner [34]. Together, these observations are consistent with a more conservative physiological state in aging cambium, characterized by reduced investment in high-cost growth processes and altered hormonal regulation.

### 3.3. Age-Associated Phenylpropanoid Metabolism Is Consistent with Structural Reinforcement and Stress-Related Metabolic Changes

Cambial aging was associated with reduced cell division and expansion, together with increased accumulation of phenolic acids, flavonoids, and lignin-related metabolites [35]. With increasing tree age, pathways related to phenylalanine metabolism, phenylpropanoid biosynthesis, and associated secondary metabolism were consistently enriched. This pattern may reflect an age-associated shift in metabolic allocation and is consistent with potential cell-wall reinforcement and stress-related metabolic functions. However, metabolite accumulation alone does not demonstrate enhanced antioxidant, antimicrobial, or other defence functions.

This metabolic shift was biologically meaningful because the phenylpropanoid pathway supplied precursors for lignin, flavonoids, and phenolic acid synthesis, thereby linking structural reinforcement with stress buffering and defence [36,37]. Its transcriptional control by *MYB* and *NAC* regulators was central to secondary wall formation [38,39,40], and increasing evidence showed that modulation of phenylalanine and flavonoid metabolism contributed directly to tolerance phenotypes [41]. Given that the *S. japonicum* genome also showed expansion and diversification of flavonoid- and lignin-related pathways [42], the observed enhancement of phenylpropanoid-related metabolites in aging cambial-zone-enriched tissues is consistent with a maintenance-associated metabolic shift, although functional validation is required to establish their protective roles.

The incomplete synchrony between transcript abundance and metabolite accumulation was also notable. Several structural genes in the phenylpropanoid and lignin pathways were down-regulated in older trees even as related metabolites continued to accumulate. This suggested that the metabolic state of the millennial cambium was shaped not only by instantaneous transcription but also by long-term deposition, low turnover, and redistribution of metabolic flux. Comparable age-related metabolic changes have been reported in tea and other perennial systems, where age or developmental state reshaped phenylpropanoid- and flavonoid-related metabolic outputs at scales not mirrored by one-to-one transcriptional changes [43].

Importantly, no direct antioxidant or antimicrobial assays were performed in the present study. Therefore, the detected changes in phenylpropanoid-related metabolites should not be interpreted as direct evidence of enhanced defence capacity. Instead, our data primarily support an age-associated metabolic shift and potential structural reinforcement. Future studies combining functional assays with cell-type-resolved metabolomic approaches will be required to determine whether these metabolites confer protective effects and to identify the specific cell types in which they accumulate.

### 3.4. An Integrative TF–Structural Gene–Metabolite–Functional Trait Framework Associated with Cambial Aging

Age-related cambial remodelling was associated with coordinated changes in functional traits, transcripts, and metabolites through a multi-layered regulatory network. Many DETFs, particularly from the ERF, MYB-related, and NAC families, showed clear age dependence, and several hub TFs were significantly associated with structural genes and metabolites involved in cell division, hormone metabolism, and phenylpropanoid metabolism. These relationships are consistent with coordinated associations among age-related signals, regulatory factors, downstream structural genes, and metabolic traits. This is aligned with cell-type-specific cambial transcriptomics in *Arabidopsis* and the fluctuating dynamic of the *CLE-PXY-WOX* modules during cambial activity in *Populus* [12,44].

It was found that cambial proliferation and wood formation were regulated by interacting modules involving *WOX4* protein stability, the *PtrVCS2-PtrWOX4a* axis, ABA-auxin integration, JA-cytokinin crosstalk, and transcription factors such as *PagMYB31*, *PagHAM4a/PagHAM4b*, and *PagGRF15* [30,31,32,33,34,45]. In parallel, inhibitory peptide signalling such as *PtrCLE20* provided further evidence that cambial activity was maintained by balancing pro-proliferative and restrictive inputs rather than by a single master switch [46]. Against this background, the path relationships resolved by piecewise SEM suggested that tree age was associated with cambial traits both directly and indirectly through hub transcription factors, downstream structural genes, and metabolites. Thus, the age–TF–structural gene–metabolite–functional trait pathway should be interpreted as a hypothesis-generating integrative framework rather than direct evidence of a causal regulatory pathway.

In addition, the materials used for physiological, hormonal, transcriptomic, and metabolomic analyses were cambial-zone-enriched tissues rather than purified cambial cells. These samples may have included adjacent differentiating phloem and xylem cells. Therefore, the cellular origin and localization of the detected metabolites cannot be resolved by the present bulk-tissue analysis. Although all trees were sampled on the same date during the active cambial growth period, complete radial tissue continuity from outer phloem to mature xylem was not retained in all anatomical sections because of mechanical separation along the cambial plane during sample processing. Consequently, the relative contributions of adjacent phloem and xylem derivatives to cambial-zone-enriched samples cannot be completely excluded, and detailed comparisons of cambial developmental phases among age groups should be interpreted cautiously.

## 4. Materials and Methods

### 4.1. Study Site and Plant Materials

Plant specimens were sourced from Jinci, Taiyuan, Shanxi Province, China (112°26′–112°27′ E, 37°42′–37°43′ N). The ages of 112 *S. japonicum* trees were evaluated based on historical archives and ancient-tree records maintained by the Taiyuan Garden Bureau, together with field surveys of tree growth conditions and site history, and interviews with local residents familiar with the history of the ancient trees. Based on this evaluation, nine trees representing three age classes were selected for analysis: the S group (approximately 80 years old), the B group (approximately 500 years old), and the Q group (approximately 1000 years old). Each age group contained three trees, which served as biological replicates (Table S7).

### 4.2. Collection and Preservation of Vascular Cambium Samples

To minimize variation associated with sampling time and seasonal conditions, all samples from the three age groups were collected on the same day (24 June 2024). Sampling was conducted during the active cambial growth period. At the Breast-Height Diameter (DBH) level (1.3 m above the ground), a sharp chisel was employed to extract trunk specimens with dimensions of ~3 × 3 × 1–2 cm (L × W × D), which included the phloem, cambium, and outermost xylem layers [8]. After sampling, the wounds were treated with a tree-wound healing agent, and the sampled trees have been continuously monitored by the responsible management authority. Each sample was divided into two portions. For anatomical structure observation, small blocks (~1 mm^3^) were preserved in a standard FAA solution. Additionally, cambial samples were obtained by cryosectioning and stored in liquid nitrogen [7]. These nitrogen-frozen samples were maintained at −80 °C for later use in transcript-level quantification, metabolite screening, and trait evaluation.

Cryosectioning was conducted with reference to anatomical observations of the cambial region. The cambial zone was identified as the narrow layer of small, thin-walled, radially arranged cells located between the secondary phloem and differentiating xylem. Rather than applying a uniform radial thickness across age groups, the thickness of cambial-zone tissue collected from each tree was adjusted according to its anatomically defined cambial-zone width and boundaries. Accordingly, tissues used for physiological, hormonal, transcriptomic, and metabolomic analyses were designated as cambial-zone-enriched tissues and may have contained a limited proportion of adjacent differentiating phloem and xylem cells.

### 4.3. Analysis of Cambial Functional Traits

A total of 22 functional traits were measured, including two morphological traits, six anatomical traits, six physiological traits, and eight endogenous hormone indices. The full names, abbreviations, units, and calculation formulas of all traits are listed in Table S8.

For morphological traits, DBH and bark thickness were measured for each tree using a measuring tape.

Anatomical traits were examined using conventional paraffin sectioning following He [47]. After fixation in FAA for more than 24 h, samples were dehydrated, embedded, sectioned, stained, and mounted. Microscopic observations were performed using an Olympus CX31 biological microscope (Olympus Corporation, Tokyo, Japan), and the resulting images were processed through the Mingmei Optical Fiber Digital Measurement and Analysis System (V1.5.3). Measured traits included cambial cell layer number, cambial thickness, cambial cell width, sieve tube aperture, and the thickness and width of sieve tube clusters. The cambial zone was defined as the narrow region of small, thin-walled, radially arranged cells located between the inner secondary phloem and differentiating xylem. During sample collection and tissue processing, mechanical separation along the cambial plane occurred in some specimens, resulting in incomplete retention of the xylem portion in several sections.

For antioxidant assessment, superoxide dismutase (SOD) activity was assayed via nitroblue tetrazolium photoreduction, while catalase (CAT) and peroxidase (POD) activities were estimated using colorimetric and guaiacol methods, respectively. The activities of SOD, CAT, and POD were normalized to soluble protein content and expressed as U/mg protein. Additionally, the thiobarbituric acid reaction was employed to quantify MDA content. Soluble sugar and soluble protein contents were determined following previously described protocols [48,49,50,51].

The contents of IAA, GA_1_, GA_4_, GA_7_, JA, SA, and ABA were analysed according to a previously described protocol [52]. Briefly, approximately 200 mg of ground tissue was extracted overnight at 4 °C with acetonitrile. After centrifugation, the pellet was re-extracted twice with five volumes of acetonitrile, and the supernatants were combined. C18 filler (20 mg) was added to the pooled extract, followed by vortexing and centrifugation. The filtered supernatants (0.22 μm) were analyzed on a QTRAP 5500 LC/MS/MS instrument (ESI Turbo Ion-Spray) at the facilities of Wuhan ProNets Biotechnology Co., Ltd., Wuhan, China.

### 4.4. RNA Isolation, Library Preparation, and Transcriptome Sequencing Analysis

For transcriptomic profiling, total RNA from the nine cambial specimens was recovered using a TRIzol-based extraction method. cDNA libraries were then generated and sequenced on an Illumina NovaSeq platform at Sangon Biotech Co., Ltd. (Shanghai, China). Subsequently, Trinity was employed to reconstruct the transcriptome via de novo assembly, followed by the removal of redundant transcripts. The longest transcript in each cluster was retained as a unigene for downstream analyses. Gene expression levels were quantified using Salmon and normalised as transcripts per million (TPM). Differentially expressed genes (DEGs) were identified based on the criteria of |log2FC| > 1.5 and an adjusted *p* < 0.05. Functional enrichment of these DEGs, including Gene Ontology (GO) and Kyoto Encyclopedia of Genes and Genomes (KEGG) analyses, was conducted using topGO and cluster Profiler, respectively. The Cytoscape software (V3.10.1) was employed to construct and visualize the gene co-expression networks [53].

### 4.5. Metabolite Extraction, Identification, Quantification, and Data Processing

Thirty milligrams of sample powder was extracted with 1500 µL of pre-cooled 70% methanol containing internal standards. Samples were vortexed for 30 s every 30 min for a total of six times. After centrifugation, the supernatant was collected, filtered through a 0.22 μm membrane, and subjected to UPLC-MS/MS analysis.

UPLC analysis was conducted using a 1.8 μm Waters ACQUITY HSS T3 column (2.1 mm × 100 mm) at a constant 40 °C. The elution used a 0.40 mL/min flow rate and a 4 µL injection volume, with aqueous 0.1% formic acid (A) and acetonitrile-based 0.1% formic acid (B) as mobile phases. The gradient started at 5% B, reaching 75% B in 2 min and 99% B in another 2 min, before returning to initial conditions after a 1.5 min hold. Mass spectrometry (MS) was performed in ESI ± modes (range 84–1250; resolution 35,000). Key MS settings included: 320 °C ion transfer tube, 300 °C vaporizer, and 30/5 Arb gas flows (sheath/auxiliary).

Differentially accumulated metabolites (DAMs) were identified based on VIP > 1 and |log2FC| ≥ 1 criteria. Clustering analysis was carried out using the R stats package’s (version 4.3.1) kmeans function after scaling [54]. Metabolite annotation utilized the KEGG Compound and Pathway resources, with pathway enrichment determined by hypergeometric testing. The DA score was computed as (number of up-regulated DAMs—number of down-regulated DAMs)/total detected metabolites within each specific pathway.

### 4.6. RT-qPCR Analysis

RT-qPCR was conducted to validate gene expression, beginning with the synthesis of complementary DNA (cDNA) from total RNA samples using the UNIQ-10 Column Trizol kit. The quantitative PCR assays utilized SYBR Green qPCR Master Mix, with specific primers (Table S9) generated via Primer Premier 6.0 (Premier Biosoft, Palo Alto, CA, USA). To determine relative expression levels, the 2^−ΔΔCt^ approach was applied, employing GAPDH as the housekeeping gene for normalization [55].

### 4.7. Statistical Analysis

Statistical significance was determined by one-way ANOVA followed by Tukey’s post hoc test for multiple comparisons. Significance levels were set at *p* < 0.05.

To identify key structural genes associated with metabolite accumulation, redundancy analysis (RDA) was performed using significantly related DAMs as response variables and DEGs from key metabolic pathways as explanatory variables. Variables with variance inflation factors greater than 10 were excluded prior to RDA [56]. A Mantel test was conducted to explore the relationships among metabolites, as well as between metabolites and leaf functional traits. RDA and Mantel tests were conducted using the ‘vegan’ package in the R environment [57,58].

We assessed causal linkages among variables through Piecewise SEM, which was executed using the piecewise SEM suite in R [59]. Overall model fit was assessed using Fisher’s C statistic (*p* > 0.05 indicating adequate fit). Explanatory power was evaluated via R^2^ for each endogenous variable, and predictive relevance was assessed using Q^2^ derived from 10-fold cross-validation. Path coefficient significance was tested using t-tests, and effect sizes were quantified using Cohen’s f^2^. Indirect effects were examined via bootstrap resampling, with significance determined by 95% confidence intervals. Multicollinearity was diagnosed using variance inflation factors (VIF < 5).

## 5. Conclusions

This study integrated anatomical traits, phytohormone profiles, transcriptomic data, and metabolomic profiles of cambial-zone-enriched tissues from 80-, 500-, and 1000-year-old *Styphnolobium japonicum* trees. Increasing tree age was associated with reductions in cambial cell-layer number and cambial thickness, accompanied by altered phytohormone profiles and changes in the expression of genes related to cell division, cell differentiation, and secondary metabolism. In parallel, phenolic acids, flavonoids, and lignin-related metabolites showed age-dependent accumulation patterns.

Together, these findings indicate that aging in the vascular cambial region is associated with coordinated anatomical, transcriptional, and metabolic changes, including attenuation of growth-associated processes and an age-related shift in phenylpropanoid metabolism. The accumulation of phenylpropanoid- and lignin-related metabolites is consistent with potential structural reinforcement and stress-related metabolic adjustment. However, because antioxidant, antimicrobial, or other defence-related functional assays were not performed, the present results do not directly demonstrate enhanced defence capacity.

Furthermore, the samples analyzed in this study were cambial-zone-enriched tissues that may have included limited proportions of adjacent differentiating phloem and xylem cells. Therefore, the cellular origin and spatial localization of the detected metabolites cannot be resolved using the present bulk-tissue approach. In addition, the relationships identified by piecewise structural equation modeling should be interpreted as statistical associations rather than direct causal regulatory pathways. Future studies combining cell-type-resolved analyses with functional validation and multi-season sampling will be necessary to clarify the biological roles of these age-associated metabolic and structural changes in the long-term maintenance of cambial activity in ancient trees.

## Data Availability

The original contributions presented in this study are included in the article/supplementary material. Further inquiries can be directed to the corresponding author.

## Supplementary Materials

The following supporting information can be downloaded at: https://www.mdpi.com/article/10.3390/plants15152337/s1. Table S1 Statistics of differentially expressed genes (DEGs); Table S2 Differential expression statistics of cell division-related genes across three age groups of *S. japonicum*; Table S3 DEGs in cell cycle pathways and cell division, differentiation, and expansion; Table S4 DEGs in the phenylpropanoid biosynthesis pathways; Table S5 DEGs in hormone synthesis metabolic pathways; Table S6 RNA-seq expression profiles of the candidate genes validated by RT-qPCR; Table S7 Basic information of sampled *S. japonicum*; Table S8 List of vascular cambium functional characteristics of *S. japonicum*; Table S9 Primer information for RT-qPCR; Figure S1. OPLS-DA score scatter plot (supervised) for the discrimination of age groups; Figure S2. Differential abundance (DA) scores of differentially accumulated metabolites among the three comparison groups (B_vs_S, Q_vs_B, and Q_vs_S); Figure S3. Integrated KEGG pathway enrichment analysis of the combined metabolome and transcriptome data across three pairwise comparison groups. This integration jointly maps DAMs and DEGs to identify co-enriched biological pathways.

## Abbreviations

The following abbreviations are used in this manuscript:

ABA	Abscisic acid
BR	Brassinosteroid
BT	Bark Thickness
CaCL	Cambial Cell Layers
CaCT	Cambial Cell Thickness
CaCW	Cambium Cell Width
CAT	Catalase
CV	coefficients of variation
DAMs	Differentially accumulated metabolites
DBH	Diameter Breast Hight
DEGs	Differentially expressed unigenes
DETFs	Differentially expressed transcription factors
DNA	Deoxyribonucleic Acid
ETH	Ethylene
FC	Fold change
GA-1	gibberellin A1
GA-4	gibberellin A4
GA-7	gibberellin A7
IAA	Auxin
IAA/ABA	Auxin-to-abscisic acid ratio
JA	Jasmonic acid
MDA	Malondialdehyde
PCA	Principal component analysis
POD	Peroxidase
PI	plasticity index
RNA	Ribonucleic Acid
ROS	Reactive oxygen species
SA	Salicylic acid
SOD	Superoxide dismutase
SP	Soluble Protein
SS	Soluble sugar content
STD	Sieve Tube Diameter
STT	Sieve Tube Thickness
STW	Sieve Tube Width
TFs	Transcription factors
TPM	Transcripts Per Million
VIP	Variable Importance in Projection

## References

[1] Fischer U., Kucukoglu M., Helariutta Y., Bhalerao R.P. (2019). The dynamics of cambial stem cell activity. Annu. Rev. Plant Biol..

[2] Wybouw B., Zhang X., Mähönen A.P. (2024). Vascular cambium stem cells: Past, present and future. New Phytol..

[3] Piovesan G., Biondi F. (2021). On tree longevity. New Phytol..

[4] Karami O., Mueller-Roeber B., Rahimi A. (2023). The central role of stem cells in determining plant longevity variation. Plant Commun..

[5] Liu S., Xu H., Wang G., Jin B., Cao F., Wang L. (2025). Tree longevity: Multifaceted genetic strategies and beyond. Plant Cell Environ..

[6] Rossi S., Deslauriers A., Anfodillo T., Carrer M. (2008). Age-dependent xylogenesis in timberline conifers. New Phytol..

[7] Wang L., Cui J., Jin B., Zhao J., Lin J.H. (2020). Multifeature analyses of vascular cambial cells reveal longevity mechanisms in old *Ginkgo biloba* trees. Proc. Natl. Acad. Sci. USA.

[8] Han X., Gai Z., Sun J., Zhai J., Qiu C., Wu Z., Li Z. (2024). Multi characteristic analysis of vascular cambium cells in *Populus euphratica* reveals its anti-aging strategy. Plants.

[9] Jajic I., Sarna T., Strzalka K. (2015). Senescence, stress, and reactive oxygen species. Plants.

[10] Bhalerao R.P., Fischer U. (2017). Environmental and hormonal control of cambial stem cell dynamics. J. Exp. Bot..

[11] Ding W., Wang C., Mei M., Li X., Zhang Y., Lin H., Li Y., Ma Z., Han J., Song X. (2024). Phytohormones involved in vascular cambium activity in woods: Current progress and future challenges. Front. Plant Sci..

[12] Zhang J., Eswaran G., Alonso-Serra J., Kucukoglu M., Xiang J., Yang W., Elo A., Nieminen K., Damén T., Joung J. (2019). Transcriptional regulatory framework for vascular cambium development in roots. Nat. Plants.

[13] Xu H., Cao D., Feng J., Wu H., Lin J., Wang Y. (2016). Transcriptional regulation of vascular cambium activity during the transition from juvenile to mature stages in *Cunninghamia lanceolata*. J. Plant Physiol..

[14] Liu G., Wu Z., Luo J., Wang C., Shang X., Zhang G. (2023). Genes expression profiles in vascular cambium of *Eucalyptus urophylla × Eucalyptus grandis* at different ages. BMC Plant Biol..

[15] Miryeganeh M. (2022). Epigenetic mechanisms of senescence in plants. Cells.

[16] Yue C., Peng H., Li W., Tong Z., Wang Z., Yang P. (2022). Untargeted metabolomics and transcriptomics reveal the mechanism of metabolite differences in spring tender shoots of tea plants of different ages. Foods.

[17] Du W., Yang J., Li Q., Jiang W., Pang Y. (2024). *Medicago truncatula* β-glucosidase 17 contributes to drought and salt tolerance through antioxidant flavonoid accumulation. Plant Cell Environ..

[18] Mu Z., Zhang Y., Zhang B., Cheng Y., Shang F., Wang H. (2023). Intraspecific chloroplast genome variation and domestication origins of major cultivars of *Styphnolobium japonicum*. Genes.

[19] Wu M., Zhang Y., Guo P., Liu H., Xia L., Wang M., Zeng C., Wang H., Shang F. (2024). Full-length transcriptome sequencing and comparative transcriptomic analyses provide comprehensive insight into molecular mechanisms of flavonoid metabolites biosynthesis in *Styphnolobium japonicum*. Genes.

[20] Thomas H. (2013). Senescence, ageing and death of the whole plant. New Phytol..

[21] Munné-Bosch S. (2015). Senescence: Is it universal or not?. Trends Plant Sci..

[22] Pasques O., Munné-Bosch S. (2022). Physiological mechanisms underlying extreme longevity in mountain pine trees. Plant Physiol..

[23] Lindenmayer D.B., Laurance W.F., Franklin J.F. (2012). Global decline in large old trees. Science.

[24] Lindenmayer D.B., Blanchard W., Blair D., McBurney L. (2018). The road to oblivion—Quantifying pathways in the decline of large old trees. For. Ecol. Manag..

[25] Li X., Liang E., Gricar J., Prislan P., Rossi S., Čufar K. (2013). Age dependence of xylogenesis and its climatic sensitivity in Smith fir on the south-eastern *Tibetan Plateau*. Tree Physiol..

[26] Kijidani Y., Ohshiro N., Iwata D., Nagamine M., Nishiyama T., Matsumura J., Koga S. (2014). Variation of indole acetic acid (IAA) amounts in cambial-region tissues in 7- and 24-year-old sugi (*Cryptomeria japonica*) trees. J. Wood Sci..

[27] Sorce C., Giovannelli A., Sebastiani L., Anfodillo T. (2013). Hormonal signals involved in the regulation of cambial activity, xylogenesis and vessel patterning in trees. Plant Cell Rep..

[28] Smetana O., Mäkilä R., Lyu M., Amiryousefi A., Sánchez Rodríguez F., Wu M., Solé-Gil A., Gavarrón M.L., Siligato R., Miyashima S. (2019). High levels of auxin signalling define the stem-cell organizer of the vascular cambium. Nature.

[29] Tylewicz S., Petterle A., Marttila S., Miskolczi P., Azeez A., Singh R.K., Immanen J., Mahler N., Hvidsten T.R., Eklund D.M. (2018). Photoperiodic control of seasonal growth is mediated by ABA acting on cell–cell communication. Science.

[30] Tang X., Wang C., Chai G., Wang D., Xu H., Liu Y., He G., Liu S., Zhang Y., Kong Y. (2022). Ubiquitinated DA1 negatively regulates vascular cambium activity through modulating the stability of *WOX4* in *Poplar*. Plant Cell.

[31] Dai X., Zhai R., Lin J., Wang Z., Meng D., Li M., Mao Y., Gao B., Ma H.Y., Zhang B. (2023). Cell-type-specific *PtrWOX4a* and *PtrVCS2* form a regulatory nexus with a histone modification system for stem cambium development in *Populus trichocarpa*. Nat. Plants.

[32] Guo X., Li J., Li M., Zhou B., Zheng S., Li L. (2024). A molecular module connects abscisic acid with auxin signals to facilitate seasonal wood formation in *Poplar*. Plant Cell Environ..

[33] Hu M.X., Guo W., Song X.Q., Liu Y.L., Xue Y., Cao Y., Hu J.J., Lu M.Z., Zhao S.T. (2024). *PagJAZ5* regulates cambium activity through coordinately modulating cytokinin concentration and signaling in *Poplar*. New Phytol..

[34] Zhang Y., Wang L., Wu Y., Wang D., He X.Q. (2024). Gibberellin promotes cambium reestablishment during secondary vascular tissue regeneration after girdling in an auxin-dependent manner in *Poplar*. J. Integr. Plant Biol..

[35] Cui J., Li X., Lu Z., Jin B. (2024). Plant secondary metabolites involved in the stress tolerance of long-lived trees. Tree Physiol..

[36] Vogt T. (2010). Phenylpropanoid biosynthesis. Mol. Plant.

[37] Weng J.K. (2014). The evolutionary paths towards complexity: A metabolic perspective. New Phytol..

[38] McCarthy R.L., Zhong R., Ye Z.H. (2009). *MYB83* is a direct target of *SND1* and acts redundantly with *MYB46* in the regulation of secondary cell wall biosynthesis in *Arabidopsis*. Plant Cell Physiol..

[39] Mitsuda N., Iwase A., Yamamoto H., Yoshida M., Seki M., Shinozaki K., Ohme-Takagi M. (2007). *NAC* transcription factors, *NST1* and *NST3*, are key regulators of the formation of secondary walls in woody tissues of *Arabidopsis*. Plant Cell.

[40] Zhou J., Lee C., Zhong R., Ye Z.H. (2009). *MYB58* and *MYB63* are transcriptional activators of the lignin biosynthetic pathway during secondary cell wall formation in *Arabidopsis*. Plant Cell.

[41] An Z., Yang Z., Zhou Y., Huo S., Zhang S., Wu D., Shu X., Wang Y. (2024). *OsJRL* negatively regulates rice cold tolerance via interfering phenylalanine metabolism and flavonoid biosynthesis. Plant Cell Environ..

[42] Chen H., Yao X., Cao B., Zhang B., Lu L., Mao P. (2022). A chromosome-level genome assembly of *Styphnolobium japonicum* combined with comparative genomic analyses offers insights on the evolution of flavonoid and lignin biosynthesis. Ind. Crops Prod..

[43] Shi Z., Cai Y., Tian Y., Zhang Y., Shao J., Liu J., Zhang Q. (2025). Determining the influence of tree age on tea quality-related metabolites in *Camellia tachangensis* var. *Remotiserrata* by integrating metabolomic and transcriptomic analyses. Front. Plant Sci..

[44] Galibina N.A., Moshchenskaya Y.L., Tarelkina T.V., Nikerova K.M., Korzhenevskii M.A., Serkova A.A., Afoshin N.V., Semenova L.I., Ivanova D.S., Guljaeva E.N. (2023). Identification and expression profile of *CLE41/44-PXY-WOX* genes in adult *Pinus sylvestris* L. trunk tissues during cambial activity. Plants.

[45] Zhou H., Song X., Lu M.Z. (2024). Growth-regulating factor 15-mediated vascular cambium differentiation positively regulates wood formation in hybrid poplar (*Populus alba × P. glandulosa*). Front. Plant Sci..

[46] Zhu Y., Song D., Zhang R., Luo L., Cao S., Huang C., Sun J., Gui J., Li L. (2020). A xylem-produced peptide *PtrCLE20* inhibits vascular cambium activity in *Poplar*. Plant Biotechnol. J..

[47] He N., Liu C., Tian M., Li M., Hao Y., Yu G., Guo D., Smith M.D., Qiang Y., Hou J. (2018). Variation in leaf anatomical traits from tropical to cold-temperate forests and linkage to ecosystem functions. Funct. Ecol..

[48] Hong B., Zhou B., Peng Z., Yao M., Wu J., Wu X., Guan C., Guan M. (2023). Tissue-specific transcriptome and metabolome analysis reveals the response mechanism of Brassica napus to waterlogging stress. Int. J. Mol. Sci..

[49] Wang W., Rui H., Yu L., Jin N., Liu W., Guo C., Cheng Y., Lou Y. (2023). Four-chlorophenoxyacetic acid treatment induces the defense resistance of rice to white-backed planthopper *Sogatella furcifera*. Int. J. Mol. Sci..

[50] Zhang D., Xue Y., Feng N., Bai J., Ma D., Sheng Q., Cao F., Zhu Z. (2024). Physiological responses and salt tolerance evaluation of different varieties of Bougainvillea under salt stress. Plants.

[51] Lin Y., Li Y., Zheng Y., Deng Y., Liu K., Gan Y., Li H., Wang J., Peng J.W., Deng R.Z. (2025). Developing quorum sensing-based collaborative dynamic control system in *Halomonas* TD01. Adv. Sci..

[52] Wei Z., Li Y., Ali F., Wang Y., Liu J., Yang Z., Wang Z., Xing Y., Li F. (2022). Transcriptomic analysis reveals the key role of histone deacetylation via mediating different phytohormone signalings in fiber initiation of cotton. Cell Biosci..

[53] Shannon P., Markiel A., Ozier O., Baliga N.S., Wang J.T., Ramage D., Amin N., Schwikowski B., Ideker T. (2003). Cytoscape: A software environment for integrated models of biomolecular interaction networks. Genome Res..

[54] Yang C., Shen S., Zhou S., Li Y., Mao Y., Zhou J., Shi Y., An L., Zhou Q., Peng W. (2022). Rice metabolic regulatory network spanning the entire life cycle. Mol. Plant.

[55] Liu T., Su B. (2021). *Styphnolobium japonicum* (L.) Schott flower extract alleviates oxidative stress and inflammatory factors in the adjuvant-induced arthritis rat model. J. Pain Res..

[56] Shrestha N. (2020). Detecting multicollinearity in regression analysis. Am. J. Appl. Math. Stat..

[57] Huhe, Chen X., Hou F., Wu Y., Cheng Y. (2017). Bacterial and fungal community structures in *Loess Plateau* grasslands with different grazing intensities. Front. Microbiol..

[58] Li K., Yuan M., Wu Y., Pineda M., Zhang C., Chen Y.F., Chen Z.Q., Rong X.L., Turnbull J.E., Guo J. (2022). A high-fat high-fructose diet dysregulates the homeostatic crosstalk between gut microbiome, metabolome, and immunity in an experimental model of obesity. Mol. Nutr. Food Res..

[59] Lefcheck J.S. (2016). Piecewise SEM: Piecewise structural equation modelling in R for ecology, evolution, and systematics. Methods Ecol. Evol..

