# Supplementary material for: Dynamics of Functional Traits and Molecular Regulation in the Vascular Cambium Across Different Ages of Styphnolobium japonicum
Source: Plants (Basel). 2026 Jul 29;15(15):2337. doi: 10.3390/plants15152337 (PMC13467693; doi:10.3390/plants15152337)
Supplement: Supplementary file 1 [file plants-15-02337-s001.zip › plants-4429215-supplementary.pdf]

Dynamics of functional traits and molecular regulation in the vascular cambium across different ages of *Styphnolobium japonicum*

Supplementary material

Table S1 Statistics of differentially expressed genes (DEGs)

	B_vs_S	Q_vs_S	Q_vs_B
up-regulated	1381	1991	1522
down-regulated	1860	2622	3066
Total	3241	4613	4588

Table S2 Differential expression statistics of cell division-related genes across three age groups of *S. japonicum*

Gene	Mean_S	Mean_B	Mean_Q	log_2FC_ B_vs_S	P_value_ B_vs_S	Padj_ B_vs_S	log_2FC_ Q_vs_S	P_value_ Q_vs_S	Padj_ Q_vs_S	log_2FC_ Q_vs_B	P_value_ Q_vs_B	Padj_ Q_vs_B
ACTF(1)	161.77	52.13	28.83	-1.63	0.02	0.14	-2.47	0.01	0.05	-0.84	0.15	0.33
ACTF(2)	25.89	9.35	8.69	-1.37	0.00	0.06	-1.51	0.02	0.07	-0.14	0.67	0.74
ACTF(3)	214.49	97.72	36.87	-1.21	0.14	0.28	-2.50	0.01	0.06	-1.29	0.12	0.33
ACTF(4)	35.75	20.89	10.66	-0.76	0.10	0.24	-1.72	0.03	0.07	-0.96	0.12	0.33
AGD9	22.79	5.84	5.08	-1.71	0.04	0.18	-2.02	0.03	0.07	-0.30	0.58	0.67
ATK1(2)	6.61	2.62	1.64	-1.16	0.09	0.24	-1.54	0.01	0.07	-0.38	0.43	0.55
ATK1(3)	14.96	7.72	6.48	-0.92	0.10	0.24	-1.11	0.03	0.07	-0.19	0.68	0.74
ATK1(5)	1.93	0.51	0.45	-0.96	0.01	0.10	-1.04	0.02	0.07	-0.09	0.77	0.79
ATK1(7)	1.34	0.75	0.27	-0.46	0.27	0.37	-0.87	0.03	0.07	-0.41	0.30	0.45
ATK5	7.31	4.52	1.93	-0.67	0.21	0.33	-1.55	0.02	0.07	-0.87	0.14	0.33
BUB1	13.06	8.25	5.02	-0.68	0.19	0.32	-1.25	0.02	0.07	-0.57	0.25	0.41
CCNA (3)	12.51	9.15	4.75	-0.52	0.43	0.52	-1.20	0.03	0.07	-0.68	0.31	0.45
CCNB1(2)	5.60	2.80	2.19	-0.79	0.04	0.18	-1.07	0.04	0.07	-0.28	0.45	0.56
CCNB1(3)	9.54	6.91	3.32	-0.45	0.20	0.32	-1.33	0.04	0.07	-0.88	0.08	0.33
CCNB1(4)	27.76	15.72	7.89	-0.87	0.15	0.29	-1.73	0.01	0.07	-0.86	0.16	0.33
CCNB1(5)	13.80	6.64	4.89	-0.96	0.02	0.14	-1.36	0.02	0.07	-0.40	0.32	0.46

CCNB1(6)	9.87	4.90	3.94	-0.85	0.07	0.21	-1.13	0.04	0.07	-0.27	0.44	0.55
CCNB1(7)	7.87	5.01	3.17	-0.58	0.19	0.32	-1.07	0.02	0.07	-0.49	0.22	0.39
CCNB1(8)	10.14	8.45	3.04	-0.37	0.52	0.60	-1.49	0.01	0.07	-1.12	0.13	0.33
CDH1(5)	16.22	13.93	9.18	-0.28	0.54	0.62	-0.74	0.03	0.07	-0.47	0.33	0.47
EB1C(1)	7.25	1.75	2.09	-1.79	0.09	0.24	-1.48	0.04	0.07	0.31	0.70	0.75
EB1C(2)	30.79	19.11	10.56	-0.69	0.12	0.25	-1.54	0.04	0.07	-0.85	0.16	0.33
EXPA(1)	47.01	11.73	10.55	-1.98	0.02	0.14	-2.08	0.00	0.05	-0.10	0.82	0.83
EXPA(3)	193.45	33.18	45.63	-2.54	0.01	0.10	-2.13	0.02	0.07	0.41	0.36	0.48
EXPA(6)	171.19	46.52	38.37	-1.89	0.01	0.10	-2.15	0.00	0.05	-0.26	0.49	0.59
EXPA(8)	14.56	5.92	2.87	-1.19	0.02	0.14	-2.07	0.02	0.07	-0.87	0.08	0.33
EXPA(9)	21.74	25.73	7.80	0.23	0.40	0.51	-1.37	0.00	0.05	-1.59	0.00	0.10
EXPA(10)	192.36	143.22	53.71	-0.50	0.48	0.56	-1.76	0.02	0.07	-1.26	0.13	0.33
EXPA(11)	46.34	58.55	19.89	0.16	0.82	0.83	-1.16	0.02	0.07	-1.32	0.15	0.33
EXPA(12)	389.97	265.91	92.97	-0.59	0.48	0.56	-1.98	0.04	0.07	-1.38	0.12	0.33
MAD2	16.62	7.73	5.15	-1.06	0.06	0.20	-1.60	0.04	0.07	-0.54	0.33	0.47
RBL1(1)	55.62	18.51	8.86	-1.49	0.05	0.20	-2.44	0.02	0.07	-0.95	0.07	0.33
RBL1(2)	0.82	0.71	0.18	-0.11	0.63	0.69	-0.64	0.02	0.07	-0.52	0.11	0.33
SKP1	2.10	0.24	0.18	-1.26	0.06	0.20	-1.35	0.04	0.07	-0.09	0.67	0.74
WOX(1)	42.55	51.07	13.36	0.17	0.75	0.79	-1.61	0.01	0.06	-1.78	0.03	0.26
WOX(2)	110.71	99.45	28.85	-0.23	0.75	0.79	-1.82	0.03	0.07	-1.59	0.10	0.33
WOX(3)	31.61	17.38	7.74	-0.86	0.22	0.33	-1.82	0.03	0.07	-0.96	0.16	0.33
XTH(1)	38.72	46.20	1.63	0.32	0.74	0.79	-3.68	0.03	0.07	-4.00	0.01	0.23
XTH(2)	66.85	25.88	24.92	-1.39	0.04	0.18	-1.39	0.00	0.04	0.00	0.99	0.99
XTH(3)	192.28	116.78	41.49	-0.75	0.08	0.24	-2.20	0.00	0.04	-1.45	0.01	0.23
XTH(4)	21.53	12.89	7.53	-0.70	0.06	0.20	-1.45	0.03	0.07	-0.75	0.13	0.33
XTH(5)	15.07	6.53	3.50	-1.13	0.12	0.25	-1.95	0.04	0.07	-0.82	0.30	0.45
XTH(6)	182.26	163.74	38.08	-0.22	0.60	0.67	-2.23	0.00	0.01	-2.00	0.02	0.24
XTH(7)	19.46	8.58	4.80	-1.18	0.09	0.24	-1.95	0.04	0.07	-0.77	0.25	0.41
XTH(8)	35.76	34.85	7.10	-0.42	0.67	0.73	-2.39	0.04	0.07	-1.97	0.13	0.33
XTH(9)	214.84	132.67	68.99	-0.77	0.18	0.32	-1.67	0.02	0.07	-0.91	0.15	0.33

Table S3 DEGs in cell cycle pathways and cell division, differentiation, and expansion

Gene ID	Gene symbol	Gene ID	Gene symbol
TRINITY_DN17515_c0_g2	ACTF(1)	TRINITY_DN31603_c1_g2	CDK2
TRINITY_DN17515_c0_g4	ACTF(2)	TRINITY_DN21079_c1_g6	E2F3(1)
TRINITY_DN17784_c0_g2	ACTF(3)	TRINITY_DN25110_c1_g1	E2F3(2)
TRINITY_DN23119_c0_g1	ACTF(4)	TRINITY_DN11215_c0_g1	EB1C(1)
TRINITY_DN24109_c1_g3	AGD10	TRINITY_DN31508_c5_g1	EB1C(2)
TRINITY_DN27252_c0_g2	AGD11	TRINITY_DN16602_c1_g2	EXPA(1)
TRINITY_DN31067_c3_g5	AGD9	TRINITY_DN20932_c0_g6	EXPA(2)
TRINITY_DN16932_c1_g1	APC11	TRINITY_DN21480_c0_g1	EXPA(3)
TRINITY_DN15473_c0_g1	APC13	TRINITY_DN21480_c0_g2	EXPA(4)
TRINITY_DN18578_c0_g2	APC8	TRINITY_DN21480_c0_g4	EXPA(5)
TRINITY_DN30867_c1_g5	ATK1(1)	TRINITY_DN23039_c1_g2	EXPA(6)
TRINITY_DN18779_c0_g1	ATK1(2)	TRINITY_DN23039_c1_g1	EXPA(7)
TRINITY_DN30867_c1_g1	ATK1(3)	TRINITY_DN23039_c1_g3	EXPA(8)
TRINITY_DN30140_c2_g2	ATK1(4)	TRINITY_DN23475_c0_g1	EXPA(9)
TRINITY_DN12861_c0_g1	ATK1(5)	TRINITY_DN25360_c2_g7	EXPA(10)
TRINITY_DN14324_c0_g2	ATK1(6)	TRINITY_DN20932_c0_g5	EXPA(11)
TRINITY_DN30991_c1_g1	ATK1(7)	TRINITY_DN28709_c2_g2	EXPA(12)
TRINITY_DN12920_c0_g2	ATK5	TRINITY_DN28749_c2_g2	EXPA(13)
TRINITY_DN19211_c0_g1	BUB1	TRINITY_DN25004_c1_g1	IRX(1)
TRINITY_DN22479_c1_g1	CCNA(1)	TRINITY_DN25351_c0_g4	IRX(2)
TRINITY_DN26597_c1_g1	CCNA(2)	TRINITY_DN19494_c2_g1	MAD2
TRINITY_DN27197_c0_g1	CCNA(3)	TRINITY_DN26855_c2_g6	PPP2C
TRINITY_DN16949_c0_g1	CCNB1(1)	TRINITY_DN25667_c0_g2	RBL1(1)
TRINITY_DN16949_c0_g2	CCNB1(2)	TRINITY_DN25667_c0_g3	RBL1(2)
TRINITY_DN16949_c0_g5	CCNB1(3)	TRINITY_DN16464_c0_g3	SKP1
TRINITY_DN27793_c0_g1	CCNB1(4)	TRINITY_DN17088_c1_g4	WOX(1)
TRINITY_DN28228_c0_g2	CCNB1(5)	TRINITY_DN17088_c1_g6	WOX(2)
TRINITY_DN31562_c1_g1	CCNB1(6)	TRINITY_DN20748_c0_g1	WOX(3)
TRINITY_DN31562_c1_g2	CCNB1(7)	TRINITY_DN13823_c0_g1	XTH(1)
TRINITY_DN31562_c1_g3	CCNB1(8)	TRINITY_DN15188_c0_g1	XTH(2)
TRINITY_DN31354_c1_g1	CDC20	TRINITY_DN17885_c0_g3	XTH(3)
TRINITY_DN20387_c0_g1	CDC6	TRINITY_DN19771_c1_g2	XTH(4)
TRINITY_DN16668_c1_g1	CDH1(1)	TRINITY_DN20659_c1_g1	XTH(5)
TRINITY_DN16724_c0_g2	CDH1(2)	TRINITY_DN22765_c0_g1	XTH(6)
TRINITY_DN17084_c1_g1	CDH1(3)	TRINITY_DN24674_c1_g2	XTH(7)
TRINITY_DN31042_c1_g1	CDH1(4)	TRINITY_DN24674_c1_g4	XTH(8)
TRINITY_DN31899_c1_g1	CDH1(5)	TRINITY_DN28177_c2_g2	XTH(9)

Table S4 DEGs in the phenylpropanoid biosynthesis pathways

Gene ID	Gene symbol	Gene ID	Gene symbol
TRINITY_DN18295_c0_g1	4CL(1)	TRINITY_DN17967_c0_g1	E1.11.1.7(9)
TRINITY_DN18586_c0_g1	4CL(2)	TRINITY_DN19723_c0_g2	E1.11.1.7(10)
TRINITY_DN20236_c0_g4	4CL(3)	TRINITY_DN20521_c2_g4	E1.11.1.7(11)
TRINITY_DN12298_c0_g1	4CL(4)	TRINITY_DN21790_c1_g2	E1.11.1.7(12)
TRINITY_DN18586_c0_g3	4CL(5)	TRINITY_DN21790_c1_g3	E1.11.1.7(13)
TRINITY_DN25297_c0_g1	CCR	TRINITY_DN9684_c0_g1	E1.11.1.7(14)
TRINITY_DN30247_c0_g1	COMT(1)	TRINITY_DN18209_c1_g2	E1.11.1.7(15)
TRINITY_DN21047_c0_g1	COMT(2)	TRINITY_DN19723_c0_g1	E1.11.1.7(16)
TRINITY_DN21047_c0_g4	COMT(3)	TRINITY_DN28135_c0_g3	E1.11.1.7(17)
TRINITY_DN25387_c1_g1	COMT(4)	TRINITY_DN20802_c0_g1	E2.1.1.104(1)

TRINITY_DN25387_c1_g2	COMT(5)	TRINITY_DN24653_c1_g1	E2.1.1.104(2)
TRINITY_DN14489_c0_g1	CSE	TRINITY_DN20703_c2_g1	K22395(1)
TRINITY_DN14442_c0_g2	CYP73A(1)	TRINITY_DN20703_c2_g2	K22395(2)
TRINITY_DN25139_c0_g1	CYP73A(2)	TRINITY_DN28495_c0_g3	K22395(3)
TRINITY_DN25618_c1_g1	CYP84A	TRINITY_DN28495_c0_g5	K22395(4)
TRINITY_DN15745_c1_g2	E1.11.1.7(1)	TRINITY_DN29814_c1_g1	PAL(1)
TRINITY_DN15943_c0_g1	E1.11.1.7(2)	TRINITY_DN29814_c1_g5	PAL(2)
TRINITY_DN16401_c0_g1	E1.11.1.7(3)	TRINITY_DN12317_c0_g1	PRDX6
TRINITY_DN16807_c2_g2	E1.11.1.7(4)	TRINITY_DN29981_c1_g2	REF1(1)
TRINITY_DN20521_c2_g3	E1.11.1.7(5)	TRINITY_DN16945_c1_g2	REF1(2)
TRINITY_DN9048_c0_g1	E1.11.1.7(6)	TRINITY_DN29981_c1_g3	REF1(3)
TRINITY_DN11426_c0_g1	E1.11.1.7(7)	TRINITY_DN24865_c0_g1	UGT72E
TRINITY_DN16483_c1_g4	E1.11.1.7(8)	-	-

Table S5 DEGs in hormone synthesis metabolic pathways

Gene ID	Gene symbol	Gene ID	Gene symbol
TRINITY_DN28725_c1_g2	ABF(1)	TRINITY_DN18353_c0_g1	KAO(1)
TRINITY_DN21714_c0_g6	ABF(2)	TRINITY_DN18353_c0_g2	KAO(2)
TRINITY_DN15362_c0_g2	AHK2_3_4	TRINITY_DN19878_c0_g1	LOX2S(1)
TRINITY_DN23546_c0_g2	AHP(1)	TRINITY_DN22550_c3_g1	LOX2S(2)
TRINITY_DN17937_c1_g2	AHP(2)	TRINITY_DN15646_c0_g2	MFP2
TRINITY_DN17937_c1_g3	AHP(3)	TRINITY_DN17449_c2_g2	MKK4_5
TRINITY_DN15555_c1_g1	ALDH(1)	TRINITY_DN18833_c0_g1	NCED
TRINITY_DN29399_c2_g1	ALDH(2)	TRINITY_DN20713_c0_g1	NPR1
TRINITY_DN26316_c0_g2	AOC	TRINITY_DN29814_c1_g5	PAL(1)
TRINITY_DN16737_c2_g2	AOS	TRINITY_DN29814_c1_g1	PAL(2)
TRINITY_DN17819_c0_g1	ARR-A(1)	TRINITY_DN16230_c0_g1	PLA2G
TRINITY_DN18258_c0_g3	ARR-A(2)	TRINITY_DN1000_c0_g1	PP2C(1)
TRINITY_DN28757_c0_g2	ARR-A(3)	TRINITY_DN21713_c1_g1	PP2C(2)
TRINITY_DN27137_c0_g2	ARR-B(1)	TRINITY_DN10216_c0_g1	PYL(1)
TRINITY_DN25630_c0_g3	ARR-B(2)	TRINITY_DN25054_c0_g2	PYL(2)
TRINITY_DN23184_c0_g1	AUX1(1)	TRINITY_DN10737_c0_g1	SAUR(1)
TRINITY_DN21192_c0_g2	AUX1(2)	TRINITY_DN27407_c0_g1	SAUR(10)
TRINITY_DN29789_c0_g1	BSK	TRINITY_DN31685_c3_g1	SAUR(11)
TRINITY_DN15922_c9_g1	crtZ	TRINITY_DN27403_c0_g1	SAUR(12)
TRINITY_DN19909_c3_g3	CYCD3(1)	TRINITY_DN15527_c0_g2	SAUR(2)
TRINITY_DN18376_c0_g1	CYCD3(2)	TRINITY_DN20739_c1_g3	SAUR(3)
TRINITY_DN24261_c1_g1	CYCD3(3)	TRINITY_DN21510_c0_g1	SAUR(4)
TRINITY_DN12235_c0_g1	CYP85A1	TRINITY_DN22645_c1_g1	SAUR(5)
TRINITY_DN16556_c0_g1	CYP90B	TRINITY_DN13036_c0_g1	SAUR(6)
TRINITY_DN23207_c0_g2	CYP90C1(1)	TRINITY_DN20739_c0_g1	SAUR(7)
TRINITY_DN23207_c0_g1	CYP90C1(2)	TRINITY_DN20739_c1_g2	SAUR(8)
TRINITY_DN16582_c1_g5	DELLA	TRINITY_DN22645_c1_g4	SAUR(9)
TRINITY_DN19123_c1_g2	E1.3.3.6	TRINITY_DN16570_c1_g1	SNRK2
TRINITY_DN28758_c0_g2	E5.5.1.13	TRINITY_DN15279_c0_g1	TAA1
TRINITY_DN25454_c0_g1	GA20ox	TRINITY_DN15724_c0_g1	TCH4(1)
TRINITY_DN25055_c0_g1	GH3	TRINITY_DN15724_c0_g2	TCH4(2)

TRINITY_DN29258_c0_g2	GID1	TRINITY_DN15724_c0_g3	TCH4(3)
TRINITY_DN20860_c1_g3	IAA(1)	TRINITY_DN15724_c0_g4	TCH4(4)
TRINITY_DN20860_c1_g2	IAA(2)	TRINITY_DN15724_c0_g5	TCH4(5)
TRINITY_DN24209_c0_g2	IAA(3)	TRINITY_DN15724_c0_g6	TCH4(6)
TRINITY_DN28952_c0_g2	IAA(4)	TRINITY_DN18891_c1_g6	TCH4(7)
TRINITY_DN13466_c0_g1	IAA(5)	TRINITY_DN29973_c1_g2	TF
TRINITY_DN23478_c0_g1	IAA(6)	TRINITY_DN16689_c5_g1	TGA(1)
TRINITY_DN24493_c0_g1	IAA(7)	TRINITY_DN27702_c1_g2	TGA(2)
TRINITY_DN24493_c1_g2	IAA(8)	TRINITY_DN28339_c2_g1	TGA(3)
TRINITY_DN17621_c0_g3	IPT(1)	TRINITY_DN14992_c0_g1	TGA(4)
TRINITY_DN17621_c0_g4	IPT(2)	TRINITY_DN25487_c0_g2	TGA(5)
TRINITY_DN26340_c0_g1	ISS1	TRINITY_DN28899_c2_g1	TGA(6)
TRINITY_DN21657_c0_g5	JAZ	TRINITY_DN28899_c2_g4	TGA(7)
TRINITY_DN14031_c0_g1	K14486(1)	TRINITY_DN14493_c0_g1	TGL4(1)
TRINITY_DN14979_c1_g3	K14486(2)	TRINITY_DN22937_c1_g1	TGL4(2)
TRINITY_DN14979_c3_g2	K14486(3)	TRINITY_DN30409_c1_g1	ZEP
TRINITY_DN16183_c0_g1	YUCCA	-	-

Table S6 RNA-seq expression profiles of the candidate genes validated by RT-qPCR

Gene ID	Gene Name	S1	S2	S3	B1	B2	B3	Q1	Q2	Q3
TRINITY_DN23039_c1_g3	EXP10	14.30	15.00	14.37	6.24	7.25	4.27	2.93	4.15	1.52
TRINITY_DN17885_c0_g3	XTH9	198.11	183.01	195.72	133.67	134.89	81.78	35.58	40.47	48.42
TRINITY_DN22645_c1_g1	SAUR	10.29	2.75	9.33	1.80	1.49	2.06	0.73	1.01	1.86
TRINITY_DN20860_c1_g2	IAA	3.99	2.73	3.34	1.66	1.88	1.67	0.29	1.39	2.22
TRINITY_DN15943_c0_g1	E1.11.1.7	8.42	4.71	8.44	1.77	2.43	1.70	0.25	1.49	0.62
TRINITY_DN29086_c0_g1	MYB-related	64.88	31.07	64.42	24.27	25.36	14.17	8.74	11.92	18.33
TRINITY_DN17815_c0_g1	GRP2	15.18	12.39	12.14	7.58	6.93	4.72	3.44	4.74	6.69
TRINITY_DN21125_c4_g6	LOB	14.07	4.71	13.26	5.41	5.49	0.94	0.79	0.90	1.39
TRINITY_DN17088_c1_g6	WOX4	138.31	57.96	135.85	131.53	127.14	39.69	26.93	35.57	24.05
TRINITY_DN16230_c0_g1	PLA2G	17.71	7.87	16.60	10.39	8.87	3.91	2.27	3.99	2.23
TRINITY_DN16949_c0_g5	CCNB1	9.15	10.48	8.99	7.22	8.90	4.61	1.89	4.56	3.52
TRINITY_DN23039_c1_g2	EXP10	184.07	141.03	188.48	54.16	54.57	30.83	31.01	51.28	32.83

Table S7 Basic information of sampled *S. japonicum*

No.	Tree age (year)	Height (m)	Perimeter at breast height (m)	Altitude (m)	GPS coordinates	
					Longitude (E)	Latitude (N)
1	~80	14.5	1.4	780	112.2634	37.4222
2	~80	17.1	1.5	783	112.2635	37.4223
3	~80	15.2	1.5	796	112.2631	37.4224
4	~500	21.5	3.43	782	112.2648	37.4251
5	~500	20.6	3.49	783	112.2648	37.4252
6	~500	18.8	3.54	781	112.2606	37.4158
7	~1000	11.4	4.51	796	112.2630	37.4230
8	~1000	16.4	4.1	785	112.2655	37.4255
9	~1000	17	4.21	788	112.2704	37.4301

Table S8 List of vascular cambium functional characteristics of *S. japonicum*

Classification	Vascular cambium functional traits	Abbreviation	Units	Formulas
Morphological Traits (2)	Breast-Height Diameter	DBH	m	-
	Bark Thickness	BT	cm	-
Anatomical Traits (6)	Cambium Cell Layers	CaCL	-	-
	Vascular Cambial Cells Thickness	CaCT	μm	-
	Vascular Cambium Cell Width	CaCW	μm	
	Sieve Tube Diameter	STD	μm	-
	Sieve Tube Thickness	STT	μm	-
	Sieve Tube Width	STW	μm	-
Resistance physiological Traits (6)	Superoxide dismutase	SOD	U/mg	-
	Peroxidase	POD	U/mg	-
	Catalase	CAT	U/mg	
	Malondialdehyde	MDA	nmol/mg	
	Soluble sugar content	SS	mg/mg	-
	Soluble Protein	SP	mg/g	-
Hormonal Traits (8)	auxin	IAA	ng/g	-
	gibberellin A1	GA-1	ng/g	-
	gibberellin A4	GA-4	ng/g	-
	gibberellin A7	GA-7	ng/g	-
	jasmonic acid	JA	ng/g	-
	salicylic acid	SA	ng/g	-
	abscisic acid	ABA	ng/g	-
	auxin to abscisic acid ratio	IAA/ABA	-	IAA/ABA

Table S9 Primer information for RT-qPCR

Gene symbol	Primers	Primer sequences (5'--3')	Gene symbol	Primers	Primer sequences (5'--3')
EXP10	TRINITY_DN23039_c1_g3-F	GGCCCTAAAAACAGGTCCGA	LOB	TRINITY_DN21125_c4_g6-F	CAGAGTCTTTGGAGCCAGCA
	TRINITY_DN23039_c1_g3-R	CTAGGGGTCAGAGAAGGGCT		TRINITY_DN21125_c4_g6-R	AGTGAGCAGAGCTGGTGTTC
XTH9	TRINITY_DN17885_c0_g3-F	CTTGGACATTGCAGGTGCTG	WOX4	TRINITY_DN17088_c1_g6-F	TACGAGTTTTCTGGGGCCAC
	TRINITY_DN17885_c0_g3-R	GTTCCAGCAGAGTCACCCTC		TRINITY_DN17088_c1_g6-R	GCTTCCCAACACCGACAGTA
SAUR	TRINITY_DN22645_c1_g1-F	GCCACGCTTCGATCCAATTC	PLA2G	TRINITY_DN16230_c0_g1-F	TTGGCAGGCAGAACTCAGAG
	TRINITY_DN22645_c1_g1-R	CTCTGGTTCTTTGTTGGCGC		TRINITY_DN16230_c0_g1-R	ACGGCAAGAACAGGTCACAA
IAA	TRINITY_DN20860_c1_g2-F	ATCTACAGCACCAGCAACCC	CCNB1	TRINITY_DN16949_c0_g5-F	ACATGGCCTGCTACAATGGT
	TRINITY_DN20860_c1_g2-R	GCCAACATGTTGAGTGGCAG		TRINITY_DN16949_c0_g5-R	GGTGCATCGGAAGTACTGCT
E1.11.1.7	TRINITY_DN15943_c0_g1-F	AGCGTTGAGGGAATGACCAG	EXP10	TRINITY_DN23039_c1_g2-F	TATGGCCATCACTGGTGGTG
	TRINITY_DN15943_c0_g1-R	TGGCAAGGATGTCAGCACAT		TRINITY_DN23039_c1_g2-R	GCATTCTGTGGCCGTCAAAG
MYB-related	TRINITY_DN29086_c0_g1-F	CAAACGGCCTTCCAGTTTGG	DAPDH	DAPDH-F	TCGGAGTGAACGGATTTGGC
	TRINITY_DN29086_c0_g1-R	AGAGCTACACCACCAGTTGC		DAPDH-R	TGACAAGCTTCCCGTTCTCC
GRP2	TRINITY_DN17815_c0_g1-F	GGAAGGTCGTGGTGATGGTT			
	TRINITY_DN17815_c0_g1-R	ACTCAGGGCCTCCACCATAT			

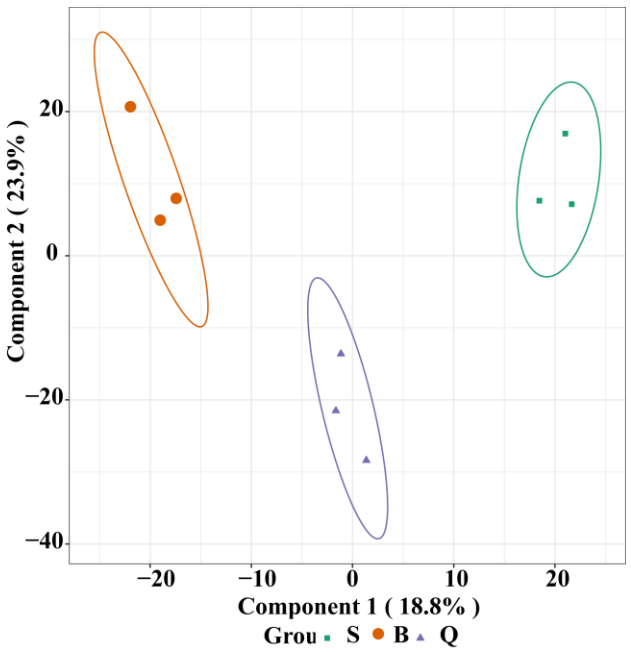

Figure S1. OPLS-DA score scatter plot (supervised) for the discrimination of age groups.

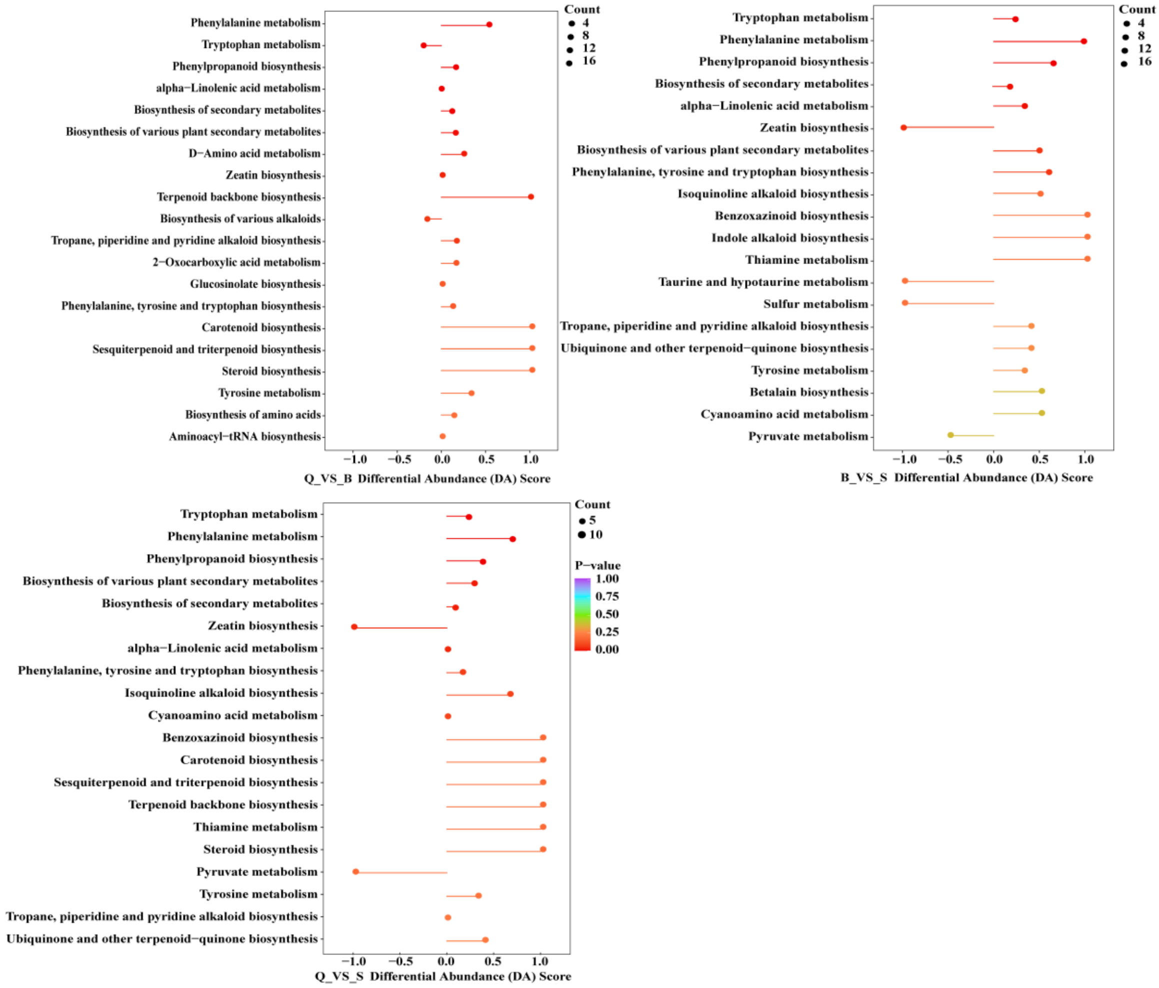

Figure S2. Differential abundance (DA) scores of differentially accumulated

metabolites among the three comparison groups (B_vs_S, Q_vs_B, and Q_vs_S).

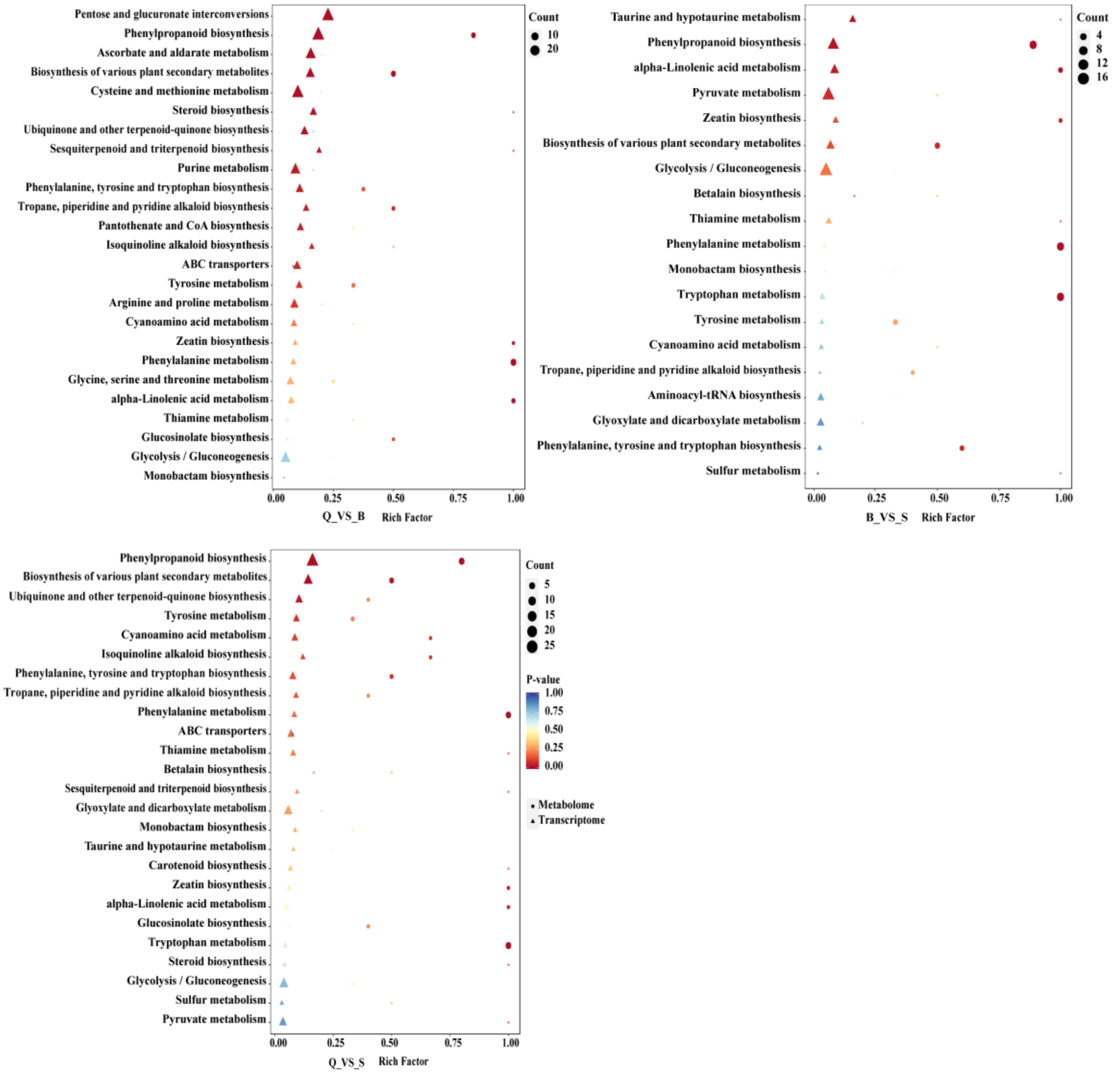

Figure S3. Integrated KEGG pathway enrichment analysis of the combined metabolome and transcriptome data across three pairwise comparison groups. This integration jointly maps DAMs and DEGs to identify co-enriched biological pathways.
